# Synthesis, Hemolytic Activity, and In Silico Studies of New Bile Acid Dimers Connected with a 1,2,3-Triazole Ring

**DOI:** 10.1021/acsomega.4c07103

**Published:** 2024-09-05

**Authors:** Grzegorz Hajdaś, Damian Kułaga, Hanna Koenig, Katarzyna Sosnowska, Lucyna Mrówczyńska, Tomasz Pospieszny

**Affiliations:** † Department of Bioactive Products, Faculty of Chemistry, Adam Mickiewicz University, Uniwersytetu Poznańskiego 8 Street, 61-614 Poznań, Poland; ‡ Department of Organic Chemistry and Technology, Faculty of Chemical Engineering and Technology, Cracow University of Technology, Warszawska 24 Street, 31-155 Kraków, Poland; § Department of Cell Biology, Faculty of Biology, Adam Mickiewicz University, Uniwersytetu Poznańskiego 6, 61-614 Poznań, Poland

## Abstract

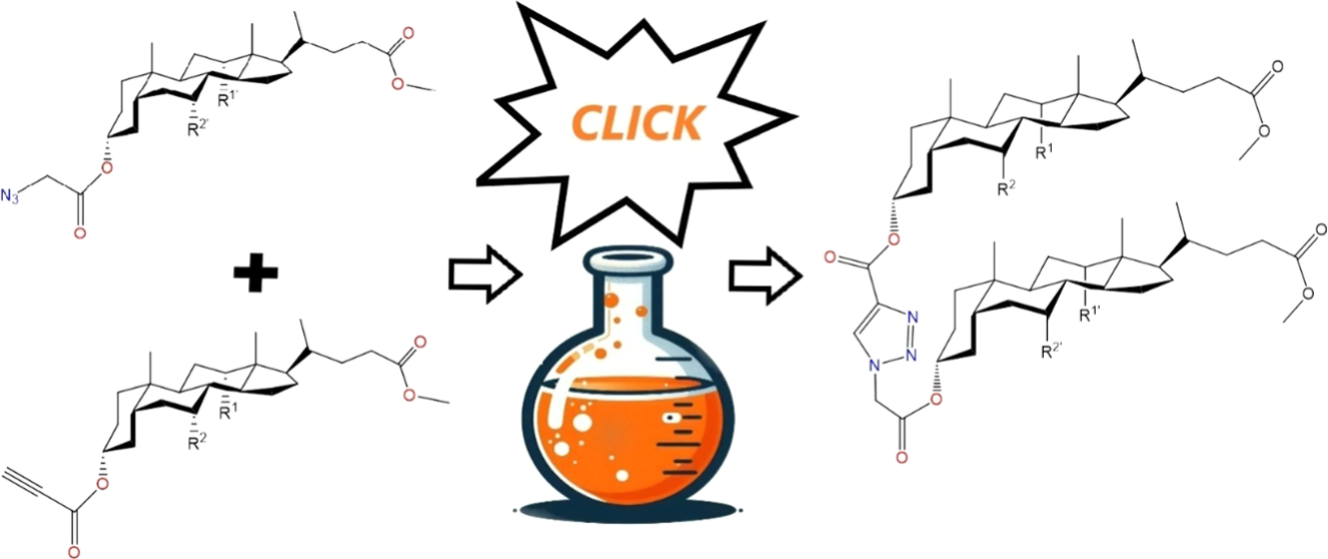

The synthesis of bile acid conjugates plays a significant role in pharmacology and organic chemistry. These complex compounds are widely studied due to their potential therapeutic applications (e.g., drug carriers or antibacterial agents) and their impact on interactions with biological target systems. It is important to determine the biological activity of the obtained conjugates with potential pharmacological applications. The research aimed to synthesize acyl conjugates of bile acids, determine the influence of acyl groups on potential antibacterial activity and evaluate the impact of conjugation on hemolytic activity. New acetyl bile acid acetyl dimers were synthesized using the “Click Chemistry” reaction, aiming to investigate their hemolytic and antibacterial activity. The structures of all compounds were confirmed through spectral analysis techniques, including ^1^H and ^13^C nuclear magnetic resonance (NMR), Fourier-transform infrared spectroscopy (FT-IR), and electrospray ionization-mass spectrometry (ESI-MS). The PM5 semiempirical method was also used to estimate the heat of formation of individual conjugates, and the prediction of activity spectra for substances (PASS) technique was used to determine the pharmacokinetic potential of compounds. Docking studies indicate that obtained conjugates have the potential ability to inhibit the biosynthesis of Lipid II and block DNA gyrase. These compounds can therefore be treated as potential candidates for antibacterial compounds. Research findings suggest that conjugating bile acids and their derivatives through 1,2,3-triazole ring, results in final products with reduced hemolytic activity.

## 1 Introduction

Steroids, as a subgroup of natural products, have long been a subject of interest in scientific research due to their potential applications across various fields, including organic chemistry and biomedicine.^1,2^ Steroids, crucial compounds found in all living organisms, play pivotal roles in many biological processes such as regulation of metabolism, immune response, and reproductive function.^4^

Bile acids—derivatives of cholesterol synthesized predominantly in the liver, form a unique subset within the steroid group.^5^ Traditionally associated with digestion and fat solubilization, bile acids have recently been identified as signaling molecules involved in glucose and energy metabolism regulation. Their amphipathic properties facilitate the solubilization and emulsification of fats during digestion.^6^ Bile acids act as signaling molecules by binding to receptors like the nuclear receptor farnesol-X-receptor (FXR) and the G-protein-coupled receptor TGR5. The distinctive structural characteristics of bile acids, including their fused four-ring core and specific hydroxyl group positions, contribute to their diverse biological activities.^7,8^ These compounds’ stereochemistry and amphiphilic nature make them suitable for various biomedical applications e.g., as antimicrobial agents.^9−15^ Bile acids are utilized in biomimetic chemistry to construct molecular receptors, supramolecular assemblies, and drug delivery systems.^16−18^ Their ability to form stable complexes with guest molecules highlights their utility in molecular recognition and host–guest interactions.^19,20^

The concept of “Click Chemistry” has revolutionized the synthesis of complex molecules, offering effective and selective reactions for chemical modification.^21,22^ The Huisgen 1,3-dipolar cycloaddition is a highly efficient technique for synthesizing disubstituted 1,2,3-triazole rings, which are resistant to oxidation, hydrolysis, and reduction. Various strategies for modifying steroids and generating bioactive derivatives with enhanced pharmacological properties have been reported, leveraging “Click Chemistry” principles.^23−26^

One promising approach involves synthesizing bile acid conjugates, which show potential for drug discovery, targeted drug delivery, and biomedical imaging due to their antifungal, antibacterial, antiviral, and anticancer properties.^27−30^ These conjugates can address the limitations of traditional steroids, such as restricted bioavailability and dose-related toxicity. The initial high hemolytic activity of bile acids can be reduced through chemical modifications. These modifications often introduce linkers like the 1,2,3-triazole ring, which is not recognized by bacteria or fungi.

Before experimental research, computational investigations are crucial to predict the chemical activity and physicochemical properties of synthesized compounds.^31,32^ Computational modeling provides valuable insights into structure–activity relationships and facilitates the rational design of bile acid derivatives with optimized pharmacological profiles. Molecular docking is an effective tool for predicting interactions between potential drugs and target bacterial proteins, accelerating the development of new antibacterial therapies.

This research aims to synthesize steroid conjugates by combining acetate derivatives of bile acids with reduced hemolytic activity and to evaluate their potential antibacterial properties. The hemolytic activity of the synthesized compounds was assessed, and molecular docking studies were conducted to predict their antibacterial efficacy. These investigations were carried out to determine the impact of conjugation on the hemolytic activity and to explore the potential of these compounds as candidates for antibacterial drug

## 2 Results and Discussion

### 2.1 Synthesis and Spectroscopic Characterization

**Scheme 1:**
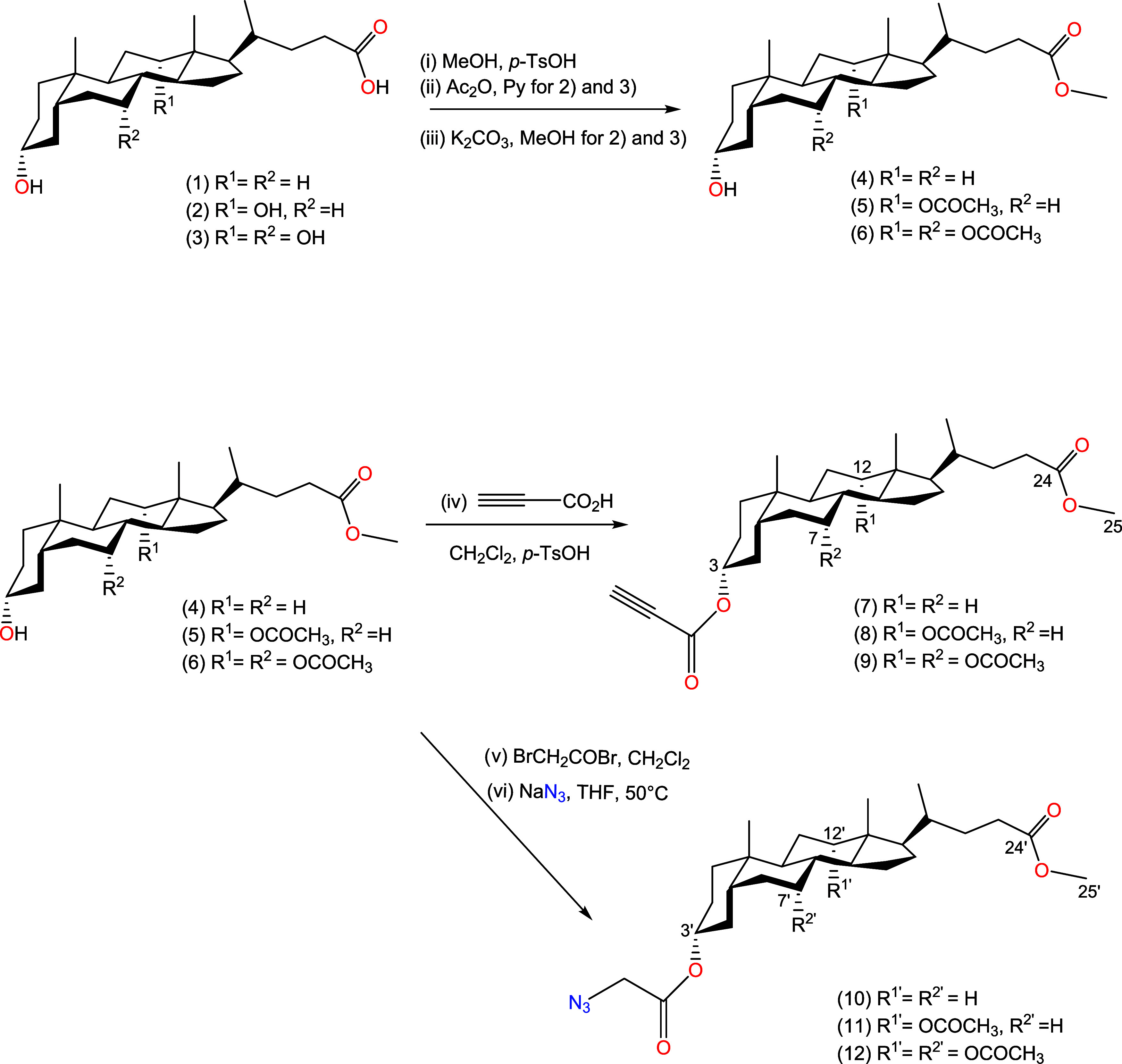
Synthesis of Propynoyl Esters of Bile Acids (**7**–**9**) Azidoacetyl Substituted Derivatives (**10**–**12**)

**Scheme 2:**
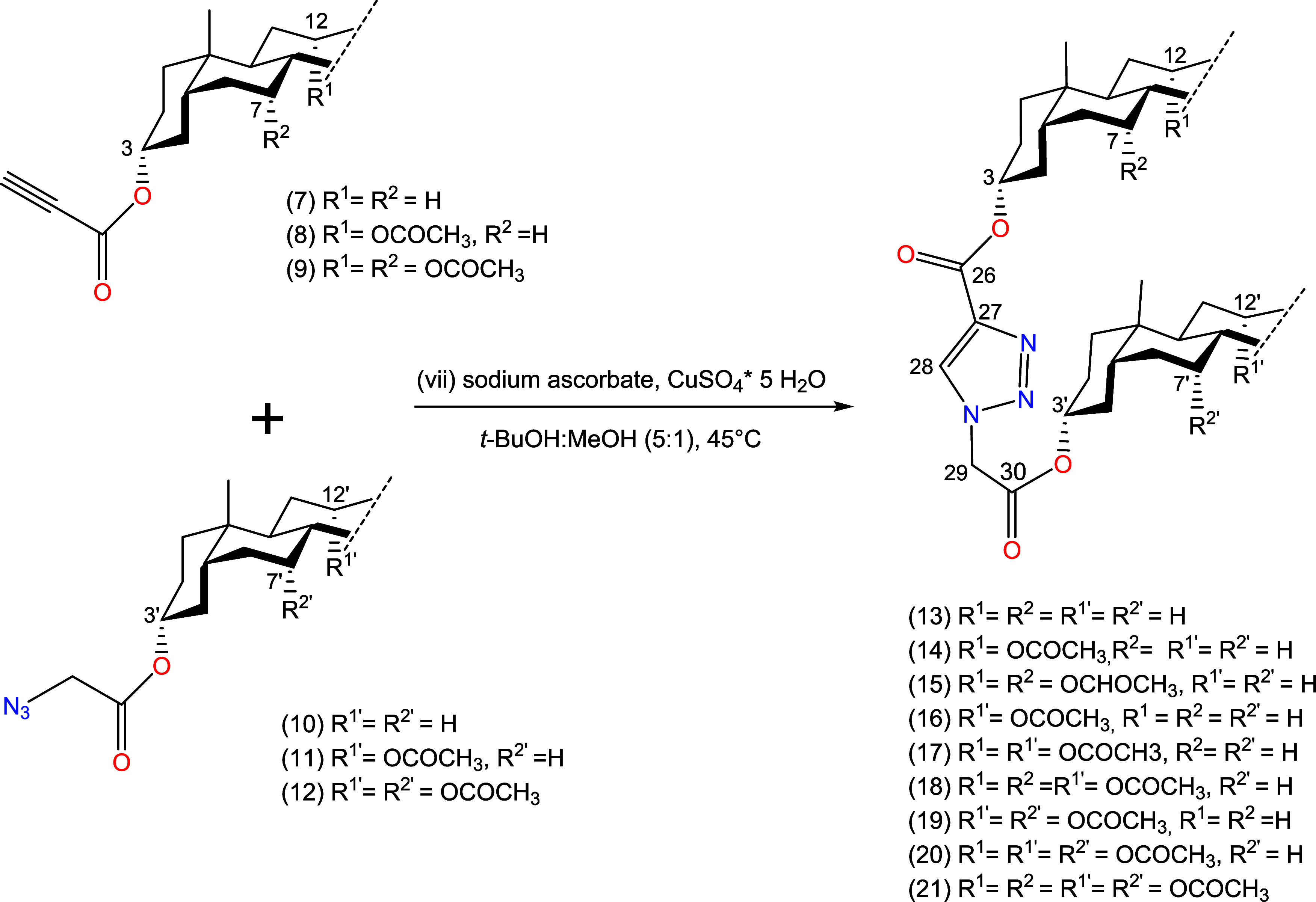
Synthesis of Dimers of Bile Acids (**13**–**21**) Linked by 1,2,3-Triazole Ring

Bile acid methyl esters and their acetoxy derivatives were successfully synthesized with decent yields, following established protocols from existing literature.^33−35^ The process involved reacting acetoxy derivatives of methyl esters of bile acids (**4**–**6**) with propiolic acid in dichloromethane and *p*-TsOH, resulting in the formation of methyl propynoyl esters (**7**–**9**) is shown in Scheme 1. To obtain the 3β-bromoacetoxy derivatives necessary for synthesizing compounds (**10**–**12**), methyl esters of bile acids (**4**–**6**) reacted with bromoacetic acid bromide in anhydrous dichloromethane. Subsequently, compounds (**10**–**12**) were synthesized from bromoacetate derivatives of bile acids via substitution reactions with NaN_3_ in THF at 50 °C, yielding azide derivatives in satisfactory quantities shown in Scheme 1. The propynoyl esters of bile acids (**7**–**9**) and azide derivatives (**10**–**12**) were used as substrates in the “Click Chemistry” reaction, conducted in the presence of CuSO_4_·5 H_2_O and sodium ascorbate in *t*-BuOH/MeOH (5:1). This led to the formation of a mixture of crude products (**13**–**21**) showed at Scheme 2, which were separated using column chromatography. The reaction yielded dimers with satisfactory yields ranging from 60 to 90%, except for compounds (**15**) (47%) and (**20**) (50%).

The basic building block of all steroids is the cyclopentanoperhydrophenanthrene ring system is not a source of many useful infrared (IR) signals. The vibrational bands corresponding to C–C bonds were notably weak and were obscured by overlapping signals in the fingerprint region. Stretching vibrations of C–H bonds merged into one broad band, for conjugate structure, between 2952 and 2867 cm^–1^. These signals are present for all compounds (**13**–**21**). The symmetric carbonyl group ν(C=O) stretching vibration induces characteristic bands at 1736–1741 cm^–1^ in the Fourier-transform-IR (FT-IR) spectrum, serving as essential signals for all products. Moreover, strong characteristic signals in the region 1247–1215 cm^–1^ are present, which are assigned to the ν(C–O). Compound (**13**), which is a derivative of lithocholic acids does not contain an acetate group in its molecule. Consequently, its FT-IR spectrum exhibits an absence of signals attributed to the ν(C=O) and ν(C–O) functional groups.

**Figure 1:**
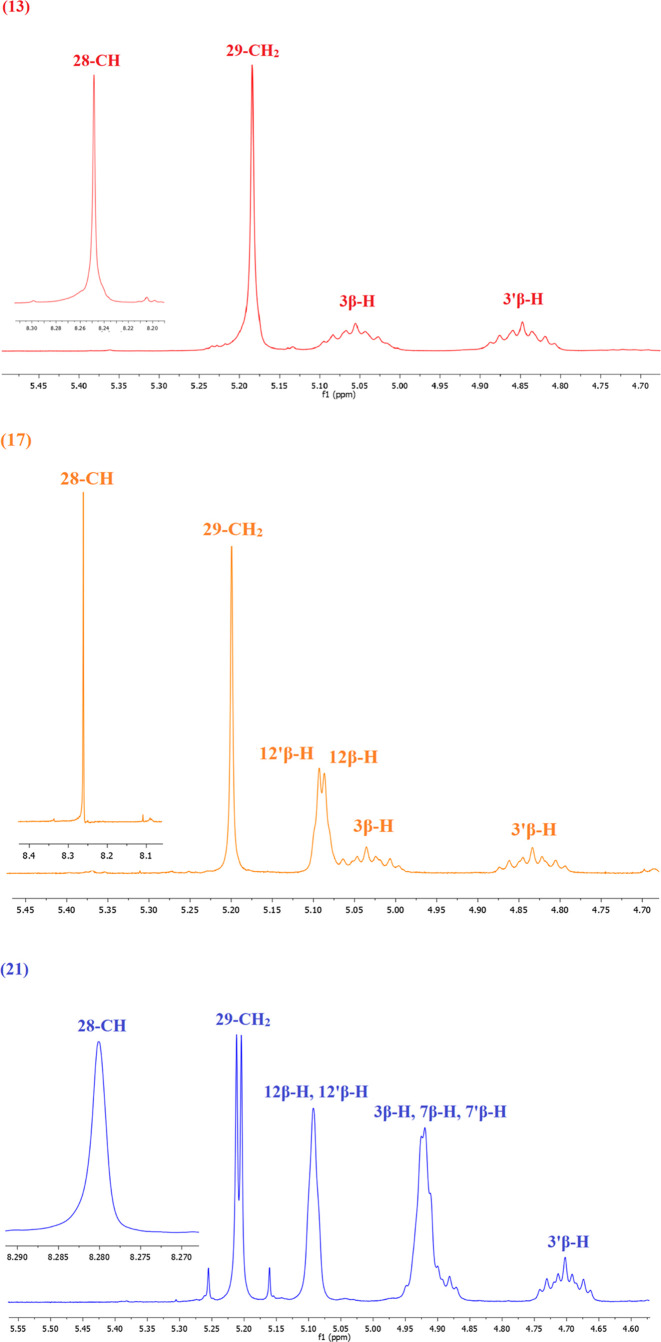
^1^H NMR spectra in the region of 4.60–8.50 ppm for the most characteristic signals of compounds (**13**, **17**, **21**).

The most characteristic signals of compounds (**13**–**21**) in the range of 3.90–8.50 ppm in the ^1^H NMR spectra are shown in Figure 1.

The diagnostic proton signals of the triazole ring 28-CH in all bioconjugates (**13**–**21**), featuring a 1,2,3-triazole ring are manifested as a singlet approximately at 8.28–8.25 ppm. The protons from the methylene groups 29-CH_2_ directly bonded to the triazole ring exhibit signals at approximately 5.21–5.18 ppm. However, in the case of compounds (**19**–**21**) (methyl cholate azidoacetate derivatives), these signals appear as a double singlet at 5.21 ppm.

All compounds (**13**–**21**) showed signals originating from 3β-H and 3β′-H protons. In the case of compounds derived from deoxycholic or cholic acids, signals originating from the following protons 12β-H/12′β-H (**14**–**21**) and 7β-H/7′β-H (**15**, **18**, **19**–**21**) were also present.

Compounds (**13**) and (**14**), a series of products from azidoacetate derivatives of lithocholic acid, had a 3′β-H signal in the form of a multiplet at 4.89–4.81 ppm. 3β-H signals occurred as multiplets with values ranging from 5.10 to 5.03 (**13**), 5.06 and 4.99 (**14**). Compound (**14**) also showed a signal from the 7β-H proton as a singlet at 5.08 ppm. In the case of compound (**15**), the 12′β-H proton was visible in the form of a distinct singlet at 5.09 ppm, however, the signals from 7′β-H, 3β-H, 3′β-H overlapped, creating a multiplet at 4.96–4.80 ppm.

Compounds (**16**) and (**17**), a series of products from azidoacetate derivatives of deoxycholic acid, had a 3′β-H signal in the form of a multiplet at 4.87–4.79 ppm. Compound (**17**) also showed signals from the 3β-H proton at 5.06–4.99 ppm in the form of a multiplet. Moreover, the signals from 12β′-H and 12β-H were visible as singlets at 5.09 and 5.08 ppm. In the case of compound (**16**), signals from 12′β-H and 3β-H overlapped forming a multiplet at 5.09–5.01 ppm. Signals from 12β-H and 12′β-H protons in compound (**18**) overlapped giving a singlet signal at 5.09 ppm. In addition, the signal from 7′β-H, 3β-H, and 3′β-H also overlapped, creating a multiplet at 4.94–4.76 ppm (**18**).

In the case of compounds (**19**–**21**), which are a series of products from azidoacetate cholate derivatives, all signals from the 3′β-H group were present in the form of a multiplet at 4.74–4.66 ppm. In compound (**19**), 12′β-H and 7′β-H protons gave clear singlet and double singlet signals at 5.09 and 4.92 ppm corresponding. Compounds (**20**–**21**) had a signal at 5.09 ppm from overlapped 12β-H and 12′β-H. In the case of compound (**20**), a multiplet from 3β-H at 5.05–4.98 ppm and a double singlet from 7β-H and 7′β-H at 4.92 ppm were visible. to 12′β-H and 7′β-H, respectively. The multiplet at 5.07–5.00 ppm was a signal originating from 3β-H. Similarly to the above, in compound (**21**) the signals from the 12β-H and 12′β-H protons overlapped to form a double singlet at 5.09. The signal at 4.94–4.86 ppm was a multiplet created by the overlap of signals from 3β-H, 7β-H, and 7′β-H.

The 25-CH_3_ and 25′-CH_3_ groups were visible as a signal at 3.67 ppm as singlet (**13**–**18**, **21**) or double singlet (**19**, **20**). The protons of the 12α-CO_2_CH_3_ group showed signals at 2.12–2.08 ppm for compounds (**14–15**, **17**, **18**, **20–21**). Similarly, signals coming from protons of 12′α-CO_2_CH_3_ were visible at 2.11–2.08 ppm for (**16**–**21**). On the other hand, protons of the 7α-CO_2_CH_3_ and 7′α-CO_2_CH_3_ groups gave signals at 2.21 and 2.15–2.14 ppm for the conjugates (**15**, **18**, **21**) and (**19**–**21**) respectively.

Two hydrogen singlets in the range 0.73–0.65, 0.93–0.91 and characteristic doublets at 0.93–0.81 ppm are assigned to 18′-CH_3_, 19′-CH_3_, and 21′-CH_3_, respectively. Similarly, singlets in ranges 0.74–0.65, 0.95–0.94 and doublets at 0.93–0.80 were assigned to 18-CH_3_, 19-CH_3_, and 21-CH_3_.

The ^13^C NMR spectra of conjugates (**19**–**21**) exhibit distinct signals at 12.4–12.2, 23.2–22.4, and 18.3–17.5 ppm, corresponding to 18′-CH_3_, 19′-CH_3_, and 21′-CH_3_, respectively. Moreover, signals originating from the group moiety range from 12.4–12.1 ppm for 18-CH_3_, 23.2–22.6 ppm for 19-CH_3_, and 18.2–17.5 ppm for 21-CH_3_. However, the resonance of the carbonyl groups of the 3α-acetoxy moiety (C26) is observed at 160.2–160.0 ppm. Conversely, the carboxyl group of the acetoxy moiety (C30) resonates at 165.1–165.1 ppm, 170.7–170.6 ppm (12α-OCOCH_3_), and 170.8 ppm (7α-OCOCH_3_) 170.4–170.2 ppm (12′α-OCOCH_3_) and 170.4 ppm (7′α-OCOCH_3_). Alternatively, the carbon atoms of the C(24)=O and C(24′)=O groups produce signals in the range of 174.8–174.5 and 174.7–174.5 ppm. The diagnostic signals for the C(27) and C(28) atoms within the 1,2,3-triazole ring are observed at 141.1 ppm and 129.2–128.8 ppm, respectively.

### 2.2 PM5 Calculations

**Figure 2:**
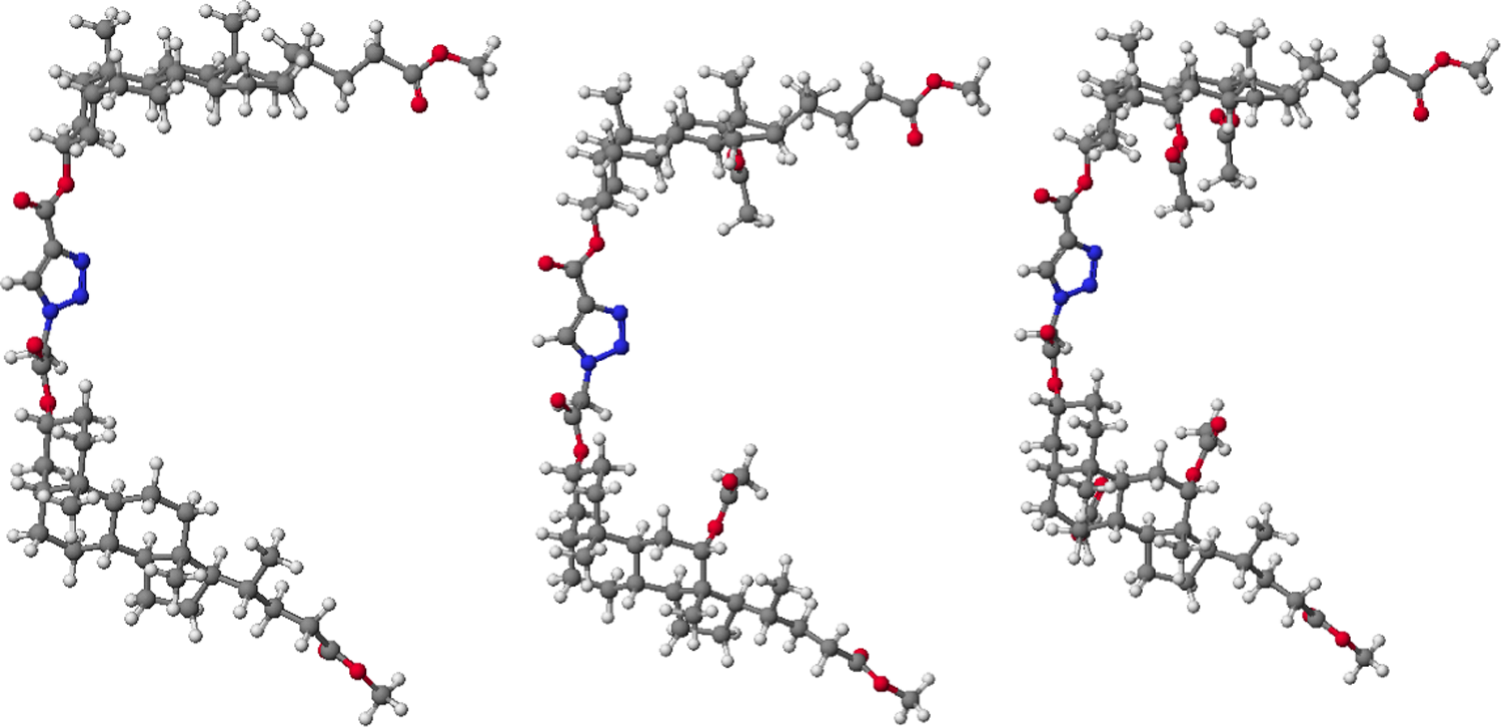
Molecular models of (**13**) (left), (**16**) (middle), and (**21**) (right) were calculated by the PM5 method.

The PM5 semiempirical calculations were performed using the WinMopac 2003 program. The final heat of formation (HOF) for dimers of bile acids linked 1,2,3-triazole ring is presented in Table 1. The molecular models of representative compounds are shown in Figure 2.

**Table 1:** Heat of Formation (HOF) [kcal/mol] of Azidoacetate Bile Acids Derivatives (**10**–**12**) and Conjugates (**13**–**21**)^a^

compounds	HOF [kcal/mol]	ΔHOF [kcal/mol]
(**10**)	–192.5621	
(**11**)	–278.4880	
(**12**)	–356.3252	
(**13**)	–453.9492	–261.3871
(**14**)	–540.5013	–347.9392
(**15**)	–613.4933	–420.9312
(**16**)	–540.0357	–261.5477
(**17**)	–626.4624	–347.9744
(**18**)	–711.7935	–433.3055
(**19**)	–617.7925	–261.4673
(**20**)	–704.2530	–347.9278
(**21**)	–789.6462	–433.3210

a ΔHOF = HOH_(**13**–**21**)_ – HOF_(**10**–**12**)_.

As evident, the end products (**13**–**21**) exhibit lower HOF values compared to their substrates. The highest HOF values are observed for the compound (**13**), while the lowest is for (**21**). The presence of hydroxyl groups in the bile acid structure, as well as their substitution by acetate groups, affects the determinant value of the HOF. Moreover, the incorporation of OAc groups facilitates the formation of stable host–guest complexes through intramolecular hydrogen bonds. Consequently, an increase in the number of OAc groups in the bile acid skeleton results in a decrease in the HOF value. These complexes can be stabilized via hydrogen bonding or electrostatic interactions stemming from the OAc groups within the bile acid molecule.

### 2.3 Prediction of Activity Spectra for Substances

The potential pharmacological activities of the synthesized bioconjugates (**13**–**21**) were determined based on a computer-aided drug discovery approach using the in silico prediction of activity spectra for substances (PASS) program.^36−40^ Biological activity predicted for a potential compound with the highest probability (focal activities) has also been selected and presented in Table 2. The most frequently predicted types of biological activity are cryoprotectant and hypolipemic. On the other hand, conjugates (**18**, **20**–**21**) have an antiviral activity and (**13**–**15**, **19**, **21**) Adenomatous polyposis inhibitor activity.

**Table 2:** Probability “To Be Active” (PA) Values for the Predicted Biological Activity of Dimmers (**13**–**21)**

compounds	(**13**)	(**14**)	(**15**)	(**16**)	(**17**)	(**18**)	(**19**)	(**20**)	(**21**)
PA > 0.7									
acylcarnitine hydrolase inhibitor	0.85	0.82	0.82	0.82	0.84	0.84	0.82	0.84	0.85
alkylacetylglycerophosphatase inhibitor	0.81	0.75		0.75	0.78	0.72		0.72	
alkenylglycerophosphocholine hydrolase inhibitor	0.76				0.72				
antieczematic	0.75	0.71		0.71					
glyceryl-ether monooxygenase inhibitor	0.72	0.76	0.80	0.76	0.77	0.80	0.80	0.80	0.80
adenomatous polyposis treatment	0.71	0.70	0.70	0.70			0.70		0.71
cholesterol antagonist	0.71								
cytoprotectant	0.70		0.73			0.71	0.73	0.71	0.70
hypolipemic			0.78			0.77	0.78	0.77	0.76
biliary tract disorders treatment			0.73			0.72	0.73	0.72	0.73
antiviral (influenza)						0.71		0.71	0.72

### 2.4 Hemolytic Activity

**Figure 3:**
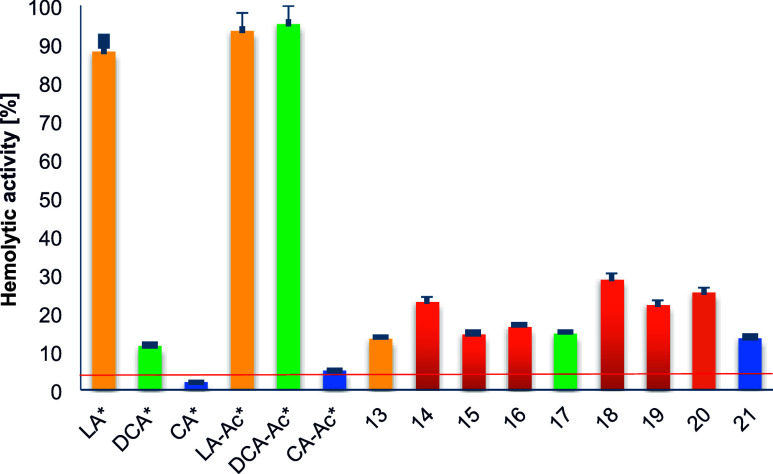
Hemolytic activity of compounds tested (0.1 mg/mL) after 1 h incubation at 37 C. A hemolysis degree higher than 5% indicates the cell membrane-perturbing activity of compounds. PBS—negative control, no hemolysis. * Adapted from data in ref (46).

Bile acids exhibit different effects on the molecular structure of cell membranes dependent on their chemical structure and concentration.^41,42^ Hydrophobic bile acids like lithocholic and deoxycholic acid provoke hemolysis in red blood cells (RBC) in a dose-dependent manner by enhancing the cell membrane permeability to ions.^43^ Conversely, at sublytic concentrations, bile acid molecules serve to stabilize the lipid bilayer of cell membranes and modulate the activity of membrane proteins such as MRP1.^44,45^ Considering the amphiphilic properties of bile acids and their derivatives, it is essential to evaluate their hemolytic activity before any biomedical uses Therefore, all compounds underwent in vitro evaluation for cytotoxicity using a hemolytic assay with human RBC. Based on literature data and the results obtained, it can be concluded that the dimerization of acetyl derivatives of bile acids results in a reduction in hemolytic activity.^46^ In the case of lithocholic (LA) and cholic acid (CA), it can be seen that the result of acylates to derivatives LA-Ac and CA-Ac is a slight increase in hemolytic activity. It is worth noting, however, that LA’s activity is much higher than the tested deoxycholic acid (DA) and CA. Acylation of DA increases its hemolytic activity to a value of 90%. For all compounds obtained, the hemolytic activity value was 13–28%, as shown in Figure 3. LA-Ac and DA-Ac conjugation with other acetyl bile acid derivatives results in obtaining dimers with lower hemolytic activity than the initial one. Only in the case of CA-Ac derivatives, an increase in hemolytic activity by 10–20% can be observed when conjugates with other acetyl derivatives. The above conclusions are particularly visible for compounds (**13**), (**18**), and (**21**) which are dimers of acetyl derivatives of lithocholic, deoxycholic and cholic acids (marked with the same color as the substrates in Figure 3).

### 2.5 Molecular Docking

In the modern world, the growing threat from antibiotic-resistant bacteria poses an urgent need for scientists and researchers to discover new chemical compounds with antibacterial properties. Drug-resistant bacteria are becoming more common, which makes existing treatments less effective. Therefore, the search for new compounds with antibacterial activity is becoming a key goal of scientists around the world., It is known that steroid derivatives may have antibacterial activities.^47^

Three representative compounds were selected for virtual screening: (**13**), (**17**), and (**21**). These are dimers of lithocholic, deoxycholic and cholic acids respectively, which additionally differ in the number of acetyl groups present. The presence or absence of these groups may therefore influence the ability to dock to selected active sites. Numerous mechanisms of antibacterial action exist. For example, antibacterial glycopeptides show activity by inhibiting Lipid II of bacteria.^47^ Lipid II plays a crucial role in bacterial cell wall synthesis as it serves as a precursor molecule for the formation of peptidoglycan, an essential component of bacterial cell walls. Peptidoglycan provides structural integrity and protection to bacterial cells, making the biosynthesis of Lipid II a vital process for bacterial survival and proliferation. In turn, fluoroquinolones, by blocking DNA gyrase, effectively destroy Gram-positive bacteria.^47,48^ DNA gyrase, which belongs to topoisomerases, is a key enzyme involved in many life processes of bacteria, including replication, transcription and recombination of bacterial DNA. It is composed of two subunits—A (responsible for cell division processes) and B (responsible for ATP hydrolysis).^49^

Due to literature reports confirming the antibacterial activity of steroids, molecular docking was performed.^47,48^ Docking involved activity to inhibit Lipid II and DNA gyrase of compounds (**13**), (**17**), and (**21**).

To assess the antibacterial activity of selected compounds, the first step is To assess the arrangement and type of interactions of a typical representative of fluoroquinolones–ciprofloxacin in the GyrB binding pocket (PBD: 3U2D).^50^ Then compare them with the arrangement and type of interactions created by the tested compounds. Docked ciprofloxacin (docking score −7.169 kJ/mol) forms stable hydrogen bond interactions with Ser129 (2.68 Å), while protonated piperazine forms a salt bridge with Glu58 (3.86 Å) and an additional hydrogen bond also with Glu58 (2.95 Å) and Asn54 (2.80 Å).

**Figure 4:**
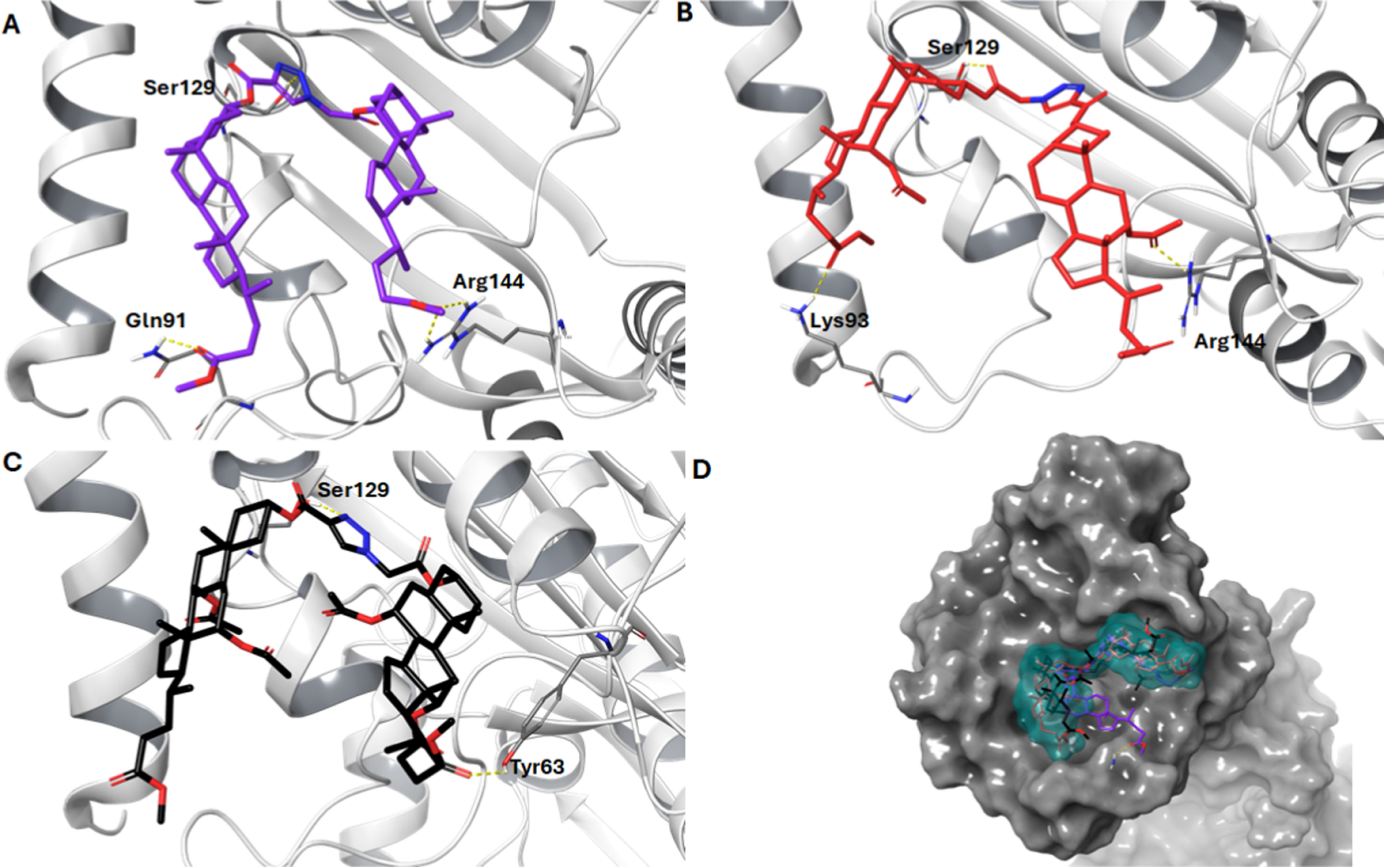
(A–C) Binding modes with hydrogen bonds of key amino acids of docked compounds (**13**)-magenta, (**17**)-red, (**21**)-black. (D) The surface of the gyrase DNA with docked compounds on. The yellow dotted line represents the hydrogen bond.

All three molecules (**13**), (**17**), and (**21**). show a similar skeletal conformation, when binding to the binding pocket of DNA gyrase. These compounds take the shape of the letter “U”, with the inflection region at the level of the triazole ring, and occupy the same space as ciprofloxacin. The results are presented in Figure 4.

For compound (**13**), the triazole forms a hydrogen interaction with Ser129 (2.91 Å) similar to that in the case of ciprofloxacin or other sterol derivatives described by Ansari et al.^49^ Both carbonyl groups of the bile acid side chain form a total of three hydrogen bonds–one double with Arg144 (2.37 and 2.34 Å) and one with Gln91 (2.17 Å). The results are presented in Figure 4A. Compound (**17**) (Figure 4B) similar to compound (**13**), it forms a hydrogen interaction with Ser129 (1.96 Å), but not through the triazole ring but through the carbonyl group at C-26 (derived from the propioloacetate derivative). Moreover, the carbonyl group present in the steroid side chain also forms hydrogen bonds with Arg144 (2.39 Å) while the carbonyl oxygen forms a hydrogen bond with Lys93 (2.12 Å). Compound (**21**) (Figure 4D) adopts a similar conformation as (**13**) and (**17**), forming hydrogen interactions with Ser129 (2.07 Å) and only one hydrogen bond between Tyr63 (2.28) with the oxygen of the bile acid side chain carbonyl group. None of the four introduced acetate groups interacts with the DNA gyrase binding pocket.

As shown in Table 3, compound (**13**) may have the best potential to inhibit DNA gyrase. It has a similar docking score and creates the largest number of hydrogen bonds. Compound (**21**), in turn, has the highest docking score and creates only two interactions with Ser129 (2.07 Å) and Tyr63 (2.28 Å).

**Table 3:** Docking Scores and Hydrogen Bonds Formed with Representative Compounds (**13**), (**17**), and (**21**)^a^

compounds	docking score (kcal/mol)	H-bond residues (distance Å)
**13**	–6.761	Ser129 (2.91 Å)
		Arg144 (2.37 Å)
		Arg144 (2.34 Å)
		Gln91 (2.17 Å)
**17**	–4.300	Ser129 (1.96 Å)
		Arg144 (2.39 Å)
		Lys93 (2.12 Å)
**21**	–3.216	Ser129 (2.07 Å)
		Tyr63 (2.28 Å)
**ciprofloxacin**	–7.169	Ser129 (2.68 Å)
		Glu58 (2.95 Å)
		Asn54 (2.80 Å)

a Ciprofloxacin is used as a reference with good antibacterial activity.

**Figure 5:**
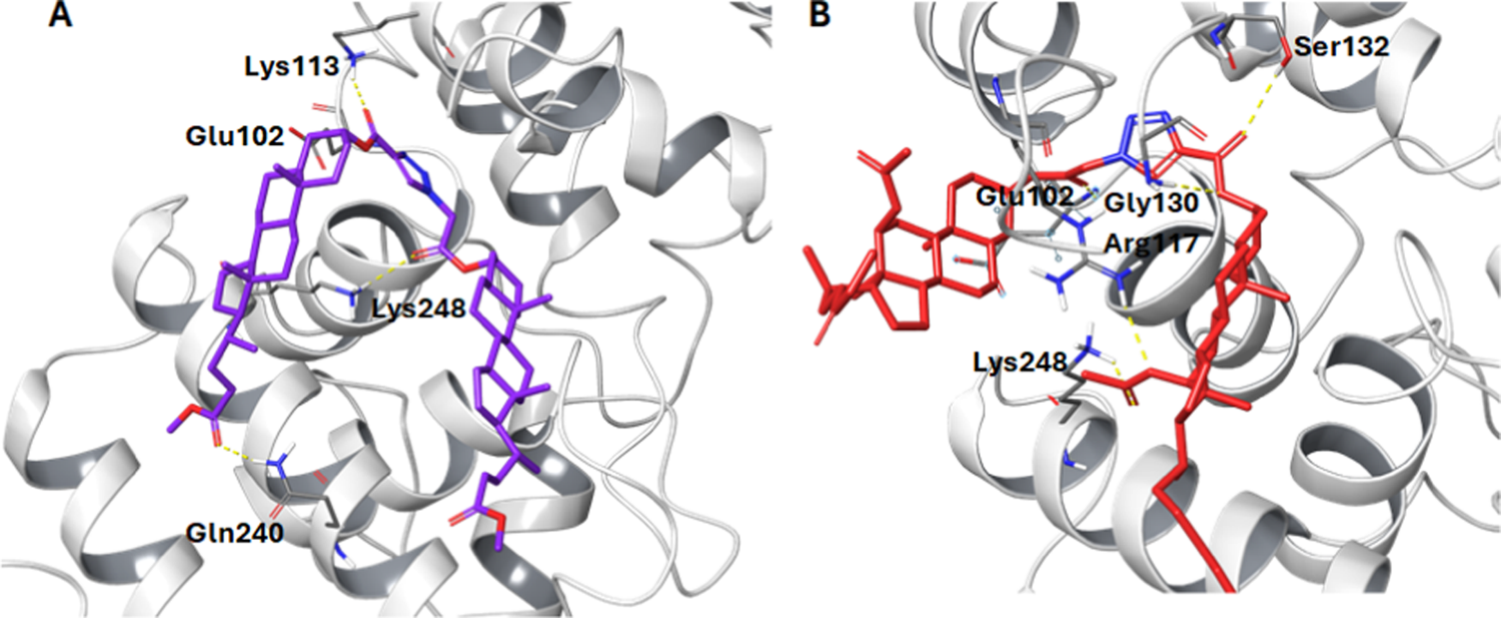
Binding modes with hydrogen bonds of key amino acids of docked compounds: (A) compound (**13**) magenta, (B) compound (**17**) red. The yellow dotted line represents the hydrogen bond.

The tested compounds were also docked to the crystal structure of *Staphylococcus aureus* membrane receptor transglycosylase (PDB: 3VMT)^51^ with parent Lipid II as a reference. The tested compounds (**13**) and (**17**), similarly to DNA gyrase, adopt a bent U-shaped conformation where the triazole ring (which is the inflection point) squeezes into the binding pocket, occupying the same space as the parent crystallized compound–LHI301 which is shown in Figure 5.

In the case of compound (**13**) (docking score −5.423 kcal/mol), the triazole forms an interaction with Glu102 (2.70 Å), while the carbonyl oxygen directly attached to the triazole through C-26 interacts with a hydrogen bond with Lys113 (2.09 Å). The carbonyl oxygen located on the other side of the triazole ring at C-30 also forms a hydrogen bond with Lys248 (2.36 Å). Furthermore, the carbonyl oxygen of the bile acid side chain forms a hydrogen bond with Gln240 (2.04 Å). For compound (**17**) (docking score −5.144 kcal/mol), the hydrogen bond with Glu102 (1.87 Å) is not formed with the triazole ring but with the carbonyl group at C-30 (from the azidoacetate derivative part). The ester group attached directly to the triazole (from the propionate derivative part), in turn, forms two hydrogen interactions with Ser132 (2.52 Å) and Gly130 (2.46 Å). The second ester group attached to the bile acid side chain (from the azide derivative part) also forms two hydrogen interactions with Arg117 (2.55 Å) and Lys248 (2.43 Å). Compound (**21**) did not dock to the crystal structure of the molecular target. Taking into account the above and analyzing the data from Table 4, both compounds (**13**) and (**17**) have the potential to exhibit antibacterial activity.

**Table 4:** Docking Scores and Hydrogen Bonds Formed with Representative Compounds (**13**) and (**17**)

compounds	docking score (kcal/mol)	H-bond residues (distance Å)
**13**	–5.423	Glu102 (2.70 Å)
		Lys113 (2.09 Å)
		Lys248 (2.36 Å)
		Glu240 (2.04 Å)
**17**	–5.144	Glu102 (1.87 Å)
		Ser132 (2.52 Å)
		Glu130 (2.46 Å)
		Arg117 (2.55 Å)
		Lys248 (2.43 Å)

## 3 Conclusions

This study presents the synthesis and chemical characterization of conjugates of bile acid derivatives connected via a 1,2,3-triazole ring (**13**–**21**). The results obtained in this study confirm that the conjugation of acetyl derivatives of lithocholic and deoxycholic acid esters causes a significant reduction in hemolytic activity of the obtained products (**13**) and (**17**). In the case of a dimer conjugate of cholic acid derivatives (**21**), an increase in hemolytic activity can be observed compared with cholic acid and its acyl derivative. The results indicate that for bile acid derivatives with high initial hemolytic activity, conjugation is a convenient method of obtaining new compounds with reduced hemolytic activity. This approach circumvents the challenge posed by the high hemolytic activity of these compounds.

Molecular docking studies prove that the compounds (**13**) and (**17**) exhibit possible binding affinity toward the protein targets of interest (3U2D and 3VMT). Inhibition of both protein’s activity could indicate the tested compound’s antibacterial properties. Docking results suggest that the antibacterial activity primarily stems from interactions associated with triazole rings or carbonyl groups, rather than acetate groups. It is therefore worth considering the possibility of other modifications of the hydroxyl groups.

Bacteria can develop resistance to commonly used antibiotics, making these drugs ineffective. This poses a serious threat to humanity. Therefore, there is an urgent need to search for new antibacterial drug candidates. The compounds obtained in this study appear valuable based on the results. This prompts further in vitro tests on pathogenic bacterial strains.

## 4 Experimental Section

### 4.1 General Procedure for the Preparation of Propiolic Esters (**7**–**9**)

The methyl esters of bile acids and their acetoxy derivatives, as well as azidoacetates or propiolic esters of bile acid were synthesized following the procedures outlined in the refs (1−3). Bile acid derivatives (**4**), (**5**), or (**6**) were dissolved in 15 mL of dichloromethane, followed by the addition of *p*-TsOH (catalytic amount) and propiolic acid (2 equiv). The reaction was carried out for 24 h at room temperature. Then the mixture was washed with cold water, extracted with chloroform, washed with water (50 mL), brine (50 mL) and dried (Na_2_SO_4_). The solvent was evaporated under reduced pressure to obtain the crude products. Products were purified by chromatography on silica gel (Merck, type 60, 70–230 mesh) with chloroform/hexane as eluent. Yields for the products (**7**–**9**): 45, 57, and 30% respectively.

### 4.2 General Procedure for the Preparation of Azidoacetates (**10**–**12**)

To obtain bromoacetate derivatives, compounds (**4**–**6**) were dissolved in 5 mL of anhydrous dichloromethane, and then subsequently, bromoacetic acid bromide was added dropwise and the reaction mixture was kept at room temperature for 24 h. Then the mixture was washed with NaHCO_3_ (5%, 20 mL), brine (100 mL) and dried over Na_2_CO_3_. The solvent was evaporated under reduced pressure to give the crude product. Products were purified by chromatography on silica gel (Merck, type 60, 70–230 mesh) with chloroform/hexane as eluent. Obtained products were dissolved in 15 mL of THF. Then, NaN_3_ (2 equiv) was added, the mixture was heated at 50 °C for 4 h. THF was evaporated, extracted with chloroform, washed with brine, and dried (Na_2_SO_4_). Yields of the products (**10**–**12**): 85, 88, and 78% respectively.

### 4.3 General Procedure for the Preparation of Bioconjugates (**13**–**21**)

Procedure for bioconjugates (**13**–**21**): Azidoacetate (**10**–**12**) was dissolved in a mixture of *t*-BuOH/MeOH (6 mL, 5:1) and compound (**7**–**9**) added. Next, CuSO_4_·5 H_2_O (3 mg, 3 mol %) and sodium ascorbate (9 mg, 20 mol %) were added to the homogeneous mixture in water (1 mL). The reaction mixture was heated at 45 °C for 1 h, then extracted with chloroform (30 mL), washed with brine (60 mL), and dried over anhydrous Na_2_SO_4_. The crude compound was purified by column chromatography on silica gel using chloroform/ethyl acetate (15:1) as an eluent and gave the products (**13**–**21**) with yields 80, 90, 47, 80, 60, 62, 70, 50, and 67% respectively.

## Supporting Information Available

^1^H and ^13^C NMR and ESI-MS spectra of bioconjugates (**13–21**) and technical and experimental specifications for PM5 calculations, hemolysis assay and molecular docking (PDF)
