# Supplementary material for: Synthesis, Hemolytic Activity, and In Silico Studies of New Bile Acid Dimers Connected with a 1,2,3-Triazole Ring
Source: ACS Omega. 2024 Sep 5;9(37):39277–86. doi: 10.1021/acsomega.4c07103 (PMC11411690; doi:10.1021/acsomega.4c07103)
Supplement: Supplementary file 1 — ao4c07103_si_001.pdf [file ao4c07103_si_001.pdf]

Suporting information for

Synthesis, hemolytic activity and *in silico* studies of new bile acid dimers connected with a 1,2,3-triazole ring

Grzegorz Hajdaś[a*], Damian Kułaga[b], Hanna Koenig[a], Katarzyna Sosnowska[c], Lucyna Mrówczyńska[c], Tomasz Pospieszny[a]

[a] *Department of Bioactive Products, Faculty of Chemistry, Adam Mickiewicz University, Uniwersytetu Poznańskiego 8 Street, 61-614 Poznań, Poland*

[b] *Department of Organic Chemistry and Technology, Faculty of Chemical Engineering and Technology, Cracow University of Technology, Warszawska 24 Street, 31-155 Kraków, Poland*

[c] *Department of Cell Biology, Faculty of Biology, Adam Mickiewicz University, Uniwersytetu Poznańskiego 6, 61-614 Poznań, Poland*

**Corresponding author: grzhaj@amu.edu.pl*

Contents:

Experimental Section

Figure S1. ^{1}H NMR spectrum of conjugate (13).

Figure S2. ^{13}C NMR spectrum of conjugate (13).

Figure S3. ESI-MS spectrum of conjugate (13).

Figure S4. ^{1}H NMR spectrum of conjugate (14).

Figure S5. ^{13}C NMR spectrum of conjugate (14).

Figure S6. ESI-MS spectrum of conjugate (14).

Figure S7. ^{1}H NMR spectrum of conjugate (15).

Figure S8. ^{13}C NMR spectrum of conjugate (15).

Figure S9. ESI-MS spectrum of conjugate (15).

Figure S10. ^{1}H NMR spectrum of conjugate (16).

Figure S11. ^{13}C NMR spectrum of conjugate (16).

Figure S12. ESI-MS spectrum of conjugate (16).

Figure S13. ^{1}H NMR spectrum of conjugate (17).

Figure S14. ^{13}C NMR spectrum of conjugate (17).

Figure S15. ESI-MS spectrum of conjugate (17).

Figure S16. ^{1}H NMR spectrum of conjugate (18).

Figure S17. ^{13}C NMR spectrum of conjugate (18).

Figure S18. ESI-MS spectrum of conjugate (18).

Figure S19. ^{1}H NMR spectrum of conjugate (19).

Figure S20. ^{13}C NMR spectrum of conjugate (19).

Figure S21. ESI-MS spectrum of conjugate (19).

Figure S22. ^{1}H NMR spectrum of conjugate (20).

Figure S23. ^{13}C NMR spectrum of conjugate (20).

Figure S24. ESI-MS spectrum of conjugate (20).

Figure S25. ^{1}H NMR spectrum of conjugate (21).

Figure S26. ^{13}C NMR spectrum of conjugate (21).

Figure S27. ESI-MS spectrum of conjugate (21).

References

Experimental Section

Lithocholic, deoxycholic and cholic acids, acetic anhydride, pyridine, propargyl alcohol, sodium azide, sodium ascorbate were purchased from Sigma-Aldrich Corporation. Solvents: chloroform, dichloromethane, toluene, hexane, t-butanol, and methanol were obtained from standard commercial sources (Merck, Fisher) and used without purification. General. IR Spectra: FT/IR-4600 type A in solid state or oil; υ in cm^{-1}. ^{1}H and ^{13}C NMR spectra: Varian Mercury 400 MHz spectrometer (Oxford, UK), operating at 400 and 101 for ^{1}H and ^{13}C, respectively; δ in ppm relative to Me_4Si as internal standard, J in Hz. Typical conditions for the H-atom spectra: pulse width 32°, acquisition time 5 s, FT size 32 K and digital resolution 0.3 Hz per point; and for the C-atom spectra: pulse width 60°, FT size 60 K and digital resolution 0.6 Hz per point, the number of scans varied from 1200 to 10.000 per spectrum. ESI-MS: Waters/Micromass (Manchester, UK) ZQ mass spectrometer equipped with a Harvard Apparatus (Saint Laurent, Canada), syringe pump; in m/z. The sample solutions were prepared in MeOH at a ca. 10^{-5} M concentration. The standard ESI-MS mass spectra were recorded at the cone voltage of 90 V**.**

1. Spectroscopic Characterization of bioconjugates (**13-21**)

1.1. 24-methoxy-24-oxo-5β-cholan-3β-yl[1-(24-metoxy-24-oxo-5β-cholan-3β-yl)]-1H-1,2,3-triazole-4-carboxylate (**13**)

Yellowish oil, yield: 80%, ^{1}H NMR (400 MHz, $CDCl_3$) δ: 8.25 (s, 1H, 28-CH), 5.18 (s, 2H, 29-CH_2), 5.10–5.03 (m, 1H, 3β-H), 4.89–4.81 (m, 1H, 3'β-H), 3.67 (s, 6H, 25-CH_3, 25'-CH_3), 0.95 (s, 3H, 19- CH_3), 0.93 (s, 3H, 19'-CH_3), 0.92 (d, *J*=6,4 Hz, 6H, 21-CH_3, 21'-CH_3), 0.65 (s, 6H, 18-CH_3,

18'-CH_3). ^{13}C NMR (101 MHz, $CDCl_3$) δ: 174.8 (C-24), 170.7 (C-24'), 165.1 (C-30), 160.1 (CO-26), 141.1 (C-27), 128.9 (C-28), 75.6 (C-3, C-3'), 56.5, 56.4, 55.9, 51.5 (C-25. C-25'), 51.2 (C-29), 42.7, 41.9, 41.9, 40.4, 40.3, 40.1, 40.0, 35.8, 35.7, 35.4, 35.3, 35.0, 34.8, 34.6, 34.5, 32.2, 32.0, 31.1, 31.0, 31.0, 28.2, 27.0, 26.9, 26.5, 26.5, 26.3, 26.2, 24.2, 24.1, 23.2 (C-19, C-19'), 20.8, 18.3 (C-21'), 18.2 (C-21), 12.0 (C-18, C-18'). FT-IR (KBr, cm^{-1}) ν_{max}: 2940, 2867, 1741, 1449, 1361. 1215, 1042, 1012, 980. ESI-MS (MeOH) *m/z* (%): 939 $[M+Na]^+$.

1.2. 24-methoxy-24-oxo-12α-acetoxy-5β-cholan-3β-yl[1-(24-metoxy-24-oxo-5β-cholan-3β-yl)]-1H-1,2,3-triazole-4-carboxylate (**14**)

Yellowish oil, yield: 90%, ^{1}H NMR (400 MHz, $CDCl_3$) δ: 8.26 (s, 1H, 28-CH), 5.19 (s, 2H, 29-CH_2), 5.08 (s, 1H, 12β-H), 5.06–4.99 (m, 1H, 3β-H), 4.89–4.81 (m, 1H, 3'β-H), 3.67 (s, 6H, 25-CH_3, 25'-CH_3), 2.12 (s, 3H, 12α-$OCOCH_3$), 0.94 (s, 3H, 19-CH_3), 0.93 (s, 3H, 19'-CH_3), 0.92 (d, *J*=6.4 Hz, 3H, 21'-CH_3), 0.81 (d, *J*=6.4 Hz, 3H, 21-CH_3), 0.73 (s, 3H, 18-CH_3), 0.65 (s, 3H, 18'-CH_3). ^{13}C NMR (101 MHz, $CDCl_3$) δ: 174.7 (C-24'), 174.6 (C-24), 170.7 (12α-O$\underline{C}$OCH_3), 165.1 (C-30), 160.2 (CO-26), 141.1 (C-27), 129.0 (C-28), 75.8 (C-12), 75.6 (C-3, C-3'), 60.2, 56.4, 55.9, 51.5 (C-25, C-25'), 51.2 (C-29), 49.3, 47.5, 45.0, 42.7, 42.0, 41.8, 40.4, 40.0, 35.7, 35.3, 34.8, 34.7, 34.6, 34.5, 34.5, 34.2, 32.2, 32.0, 31.2, 31.0, 31.0, 30.9, 30.8, 29.7, 28.1, 27.3, 27.0, 26.9, 26.6, 26.4, 26.2, 25.8, 25.8, 24.1, 23.4, 23.2 (C-19'), 23.1 (C-19), 21.5 (12α-OCO$\underline{C}H_3$), 20.8, 18.2 (C-21'), 17.5 (C-21), 14.2, 12.4 (C-18), 12.0 (C-18'). FT-IR (KBr, cm^{-1}) ν_{max}: 2947, 2868, 1739, 1542, 1450, 1379, 1245, 1170, 1099, 1043, 1020, 978. ESI-MS (MeOH) *m/z* (%): 997 $[M+Na]^+$.

1.3. 24-methoxy-24-oxo-7α,12α-diacetoxy-5β-cholan-3β-yl[1-(24-metoxy-24-oxo-5β-cholan-3β-yl)]-1H-1,2,3-triazole-4-carboxylate (**15**)

Yellowish oil, yield: 47%, ^{1}H NMR (400 MHz, $CDCl_3$) δ: 8.28 (s, 1H, 28-CH), 5.19 (s, 2H, 29-CH_2), 5.09 (s, 1H, 12'β-H), 4.96–4.80 (m, 3H, 7'β-H, 3β-H, 3'β-H), 3.67 (ds, 6H, 25-CH_3, 25'-CH_3), 2.21 (s, 3H, 7α-$OCOCH_3$), 2.08 (s, 3H, 12α-$OCOCH_3$), 0.95 (s, 3H, 19-CH_3), 0.93 (s, 3H, 19'-CH_3), 0.92 (d, *J*=6.4 Hz, 3H, 21'-CH_3), 0.81 (d, *J*=6.3 Hz, 3H, 21-CH_3), 0.74 (s, 3H, 18-CH_3), 0.65 (s, 3H, 18'-CH_3). ^{13}C NMR (101 MHz, $CDCl_3$) δ: 174.7 (C-24'), 174.5 (C-24), 170.8 (7α-O$\underline{C}$OCH_3), 170.6 (12α-O$\underline{C}$OCH_3), 165.1 (C-30), 160.2 (CO-26), 141.1 (C-27), 129.2 (C-28), 75.5 (C-12, C-3'), 75.3 (C-3), 70.6 (C-7), 56.4, 55.9, 51.5 (C-25, C-25'), 51.2 (C-29), 47.3, 45.1, 43.4, 42.7, 41.8, 41.1, 40.4, 40.0, 37.7, 35.7, 35.3, 34.8, 34.7, 34.6, 34.5, 34.5, 34.4, 32.0, 31.3, 31.2, 31.0, 31.0, 30.9, 30.7, 29.1, 28.1, 27.1, 26.9, 26.8, 26.5, 26.2, 25.7, 24.1, 23.2 (C-19'), 22.8, 22.6 (C-19), 21.8 (7α-OCO$\underline{C}H_3$), 21.7 (12α-OCO$\underline{C}H_3$), 20.8, 18.3 (C-21'), 17.5 (C-21), 12.2 (C-18), 12.0 (C-18’). FT-IR (KBr, cm^{-1}) ν_{max}: 2950, 2869, 1737, 1450, 1379, 1246, 1101, 1042, 1020, 983. ESI-MS (MeOH) *m/z* (%): 1055 $[M+Na]^+$.

1.4. 24-methoxy-24-oxo-5β-cholan-3β-yl[1-(24-metoxy-24-oxo-12α-acetoxy-5β-cholan-3β-yl)]-1H-1,2,3-triazole-4-carboxylate (**16**)

Yellowish oil, yield: 60%, ^{1}H NMR (401 MHz, $CDCl_3$) δ: 8.25 (s, 1H, 28-CH), 5.19 (s, 2H, 29-CH_2), 5.09–5.01 (m, 2H, 12'β-H, 3β-H), 4.84–4.79 (m, 1H, 3'β-H), 3.67 (ds, 6H, 25-CH_3, 25'-CH_3), 2.11 (s, 3H, 12'α-$OCOCH_3$), 0.95 (s, 3H, 19-CH_3), 0.92 (d, *J*=6.4 Hz, 3H, 21-CH_3), 0.91 (s, 3H, 19'-CH_3), 0.81 (d, *J*=6.3 Hz, 3H, 21'-CH_3), 0.73 (s, 3H, 18'-CH_3), 0.65 (s, 3H, 18-CH_3). ^{13}C NMR (101 MHz, $CDCl_3$) δ: 174.7 (C-24), 174.5 (C-24'), 170.4 (CO-12'α-O$\underline{C}$OCH_3), 165.1 (C-30), 160.0 (CO-26), 141.1 (C-27), 128.8 (C-28), 75.8 (C-12'), 75.6 (C-3, C-3'), 56.5, 56.1, 56.0, 51.5 (C-25'), 51.4 (C-25), 51.2 (C-29), 49.4, 47.6, 45.0, 42.8, 42.7, 42.0, 41.9, 41.8, 40.4, 40.3, 40.1, 35.8, 35.6, 35.4, 35.1, 34.7, 34.6, 34.6, 34.5, 34.4, 34.0, 32.2, 32.0, 31.8, 31.1, 31.0, 31.0, 30.8, 28.2, 27.3, 27.0, 26.8, 26.7, 26.6, 26.4, 26.3, 25.8, 25.6, 24.2 23.4, 23.3, 23.2 (C-19), 23.0 (C-19'), 21.3 (12'α-OCO$\underline{C}H_3$), 20.8, 18.2 (C-21), 17.5 (C-21'), 12.4 (C-18'), 12.1, 12.0 (C-18). FT-IR (KBr, cm^{-1}) ν_{max}: 2941, 2867, 1736, 1449, 1378, 1245, 1172, 1042, 1013, 974. ESI-MS (MeOH) *m/z* (%): 997 $[M+Na]^+$, 1012 $[M+K]^+$.

1.5. 24-methoxy-24-oxo-12α-acetoxy-5β-cholan-3β-yl[1-(24-metoxy-24-oxo-12α-acetoxy-5β-cholan-3β-yl)]-1H-1,2,3-triazole-4-carboxylate (**17**)

Yellowish oil, yield: 80%, ^{1}H NMR (401 MHz, $CDCl_3$) δ: 8.26 (s, 1H, 28-CH), 5.19 (s, 2H, 29-CH_2), 5.09 (s, 1H, 12'β-H), 5.08 (s, 1H, 12β-H), 5.06–4.99 (m, 1H, 3β-H), 4.87–4.79 (s, 1H, 3'β-H), 3.67 (s, 6H, 25-CH_3, 25'-CH_3), 2.12 (s, 3H, 12α-$OCOCH_3$), 2.11 (s, 3H, 12'α-$OCOCH_3$), 0.94 (s, 3H, 19-CH_3), 0.91 (s, 3H, 19'-CH_3), 0.81 (d, *J*=6.4 Hz, 3H, 21'-CH_3), 0.80 (d, *J*=6.5 Hz, 3H, 21-CH_3), 0.73 (s, 6H, 18-CH_3, 18'-CH_3). ^{13}C NMR (101 MHz, $CDCl_3$) δ: 174.6 (C-24), 174.5 (C-24'), 170.6 (12α-O$\underline{C}$OCH_3), 170.4 (12'α-O$\underline{C}$OCH_3), 165.1 (C-30), 160.1 (CO-26), 141.1 (C-27), 129.0 (C-28), 75.8 (C-12, C-12'), 75.6 (C-3, C-3'), 51.5 (C-25, C-25'), 51.2 (C-29), 49.4, 49.3, 47.6, 48.0, 45.0, 45.0, 42.0, 41.8, 35.8, 35.6, 34.8, 34.7, 34.7, 34.6, 34.5, 34.4, 34.2, 34.0, 32.3, 32.0, 31.2, 31.0, 30.8, 27.3, 27.0, 26.8, 26.6, 26.5, 25.8, 25.8, 25.6, 23.5, 23.4, 23.1 (C-19), 23.0 (C-19'), 21.5 (12α-OCO$\underline{C}H_3$), 21.3 (12'α-OCO$\underline{C}H_3$), 17.5 (C-21, C-21'), 12.4 (C-18), 12.4 (C-18'). FT-IR (KBr, cm^{-1}) ν_{max}: 2948, 2868, 1739, 1450, 1379, 1244, 1213, 1042, 1012, 977. ESI-MS (MeOH) *m/z* (%): 1054 $[M+Na]^+$, 1071 $[M+K]^+$.

1.6. 24-methoxy-24-oxo-7α,12α-diacetoxy-5β-cholan-3β-yl[1-(24-metoxy-24-oxo-12α-acetoxy-5β-cholan-3β-yl)]-1H-1,2,3-triazole-4-carboxylate (**18**)

Yellowish oil, yield: 62%, ^{1}H NMR (401 MHz, $CDCl_3$) δ: 8.28 (s, 1H, 28-CH), 5.20 (s, 2H, 29-CH_2), 5.09 (s, 2H, 12β-H, 12'β-H), 4.94–4.76 (m, 3H, 7'β-H, 3β-H, 3'β-H), 3.67 (s, 6H, 25-CH_3, 25'-CH_3), 2.21 (s, 3H, 7'α-$OCOCH_3$), 2.11 (s, 3H, 12α-$OCOCH_3$), 2.08 (s, 3H, 12'α-$OCOCH_3$), 0.95 (s, 3H, 19-CH_3), 0.92 (s, 3H, 19'-CH_3), 0.81 (d, *J*=6.3 Hz, 6H, 21-CH_3, 21'-CH_3), 0.74 (s, 3H, 18-CH_3), 0.73 (s, 3H, 18'-CH_3). ^{13}C NMR (101 MHz, $CDCl_3$) δ: 174.5 (C-24'), 174.5 (C-24), 170.8 (7α-O$\underline{C}$OCH_3), 170.6

(12α-O$\underline{C}$OCH$_3$), 170.3 (12'α-O$\underline{C}$OCH$_3$), 165.1 (C-30), 160.2 (CO-26), 141.1 (C-27), 130.8, 129.2 (C-28), 128.8, 75.8 (C-12'), 75.5 (C-12, C-3'), 75.3 (C-3), 70.6 (C-7), 51.5 (C-25, C-25'), 51.2 (C-29), 49.4, 47.6, 47.3, 45.1, 45.0, 43.4, 41.8, 41.1, 37.8, 35.6, 34.7, 34.7, 34.6, 34.5, 34.5, 34.4, 34.0, 32.0, 31.3, 31.0, 30.9, 30.8, 30.8, 30.3, 29.7, 29.1, 28.9, 27.3, 27.1, 26.8, 26.5, 25.8, 25.7, 25.6, 23.7, 23.4, 22.9 (C-19'), 22.8, 22.6 (C-19), 21.7 (7α-OCO$\underline{C}$H$_3$), 21.7 (12α-OCO$\underline{C}$H$_3$), 21.3 (12'α-OCO$\underline{C}$H$_3$), 17.5 (C-21, C-21'), 14.2, 12.4 (C-18'), 12.2 (C-18). FT-IR (KBr, cm^{-1}) ν_{max}: 2951, 2871, 1736, 1449, 1378, 1246, 1042, 1022, 985. ESI-MS (MeOH) *m/z* (%): 1113 [M+Na]$^+$, 1128 [M+K]$^+$, 1169 [M+Br]$^-$.

1.7. 24-methoxy-24-oxo-5β-cholan-3β-yl[1-(24-metoxy-24-oxo-7α,12α-diacetoxy-5β-cholan-3β-yl)]-1H-1,2,3-triazole-4-carboxylate (**19**)

Yellowish oil, yield: 67%, ^{1}H NMR (400 MHz, CDCl$_3$) δ: 8.25 (s, 1H, 28-CH), 5.20 (d, 2H, 29-CH$_2$), 5.09 (s, 1H, 12'β-H), 5.07–5.00 (m, 1H, 3β-H), 4.92 (ds, 1H, 7'β-H), 4.74–4.65 (m, 1H, 3'β-H), 3.67 (ds, 6H, 25-CH$_3$, 25'-CH$_3$), 2.15 (s, 3H, 7'α-OCOCH$_3$), 2.09 (s, 3H, 12'α-OCOCH$_3$), 0.95 (s, 3H, 19-CH$_3$), 0.93 (d, *J*=6 Hz, 6H, 21-CH$_3$, 19'-CH$_3$), 0.82 (d, *J*=6.4 Hz, 3H, 21'-CH$_3$), 0.73 (s, 3H, 18'-CH$_3$), 0.65 (s, 3H, 18-CH$_3$). ^{13}C NMR (101 MHz, CDCl$_3$) δ: 174.8 (C-24), 174.5 (C-24'), 170.4 (7'α-O$\underline{C}$OCH$_3$), 170.2 (12'α-O$\underline{C}$OCH$_3$), 165.1 (C-30), 160.0 (CO-26), 141.1 (C-27), 128.8 (C-28), 75.6 (C-3, C-12'), 75.3 (C-3'), 70.5 (C-7'), 56.4, 55.9, 51.5 (C-25'*), 51.5 (C-25*), 51.2 (C-29), 47.3, 45.0, 43.3, 42.7, 41.9, 40.8, 40.3, 40.1, 37.7, 35.8, 35.3, 35.0, 34.6, 34.4, 34.2, 32.1, 31.2, 31.1, 31.0, 31.0, 30.9, 30.7, 28.8, 28.2, 27.1, 27.0, 26.7, 26.5, 26.3, 25.5, 24.2, 23.2 (C-19), 22.8, 22.4 (C-19'), 21.6 (12'α-OCO$\underline{C}$H$_3$), 21.4 (7'α-OCO$\underline{C}$H$_3$), 20.8, 18.2 (C-21), 17.5 (C-21'), 12.2 (C-18'), 12.0 (C-18). FT-IR (KBr, cm^{-1}) ν_{max}: 2948, 2870, 1737, 1449, 1378, 1239, 1042, 1020, 965. ESI-MS (MeOH) *m/z* (%): 1055 [M+Na]$^+$, 1071 [M+K]$^+$, 1067 [M+Cl]$^-$.

1.8. 24-methoxy-24-oxo-12α-acetoxy-5β-cholan-3β-yl[1-(24-metoxy-24-oxo-7α,12α-diacetoxy-5β-cholan-3β-yl)]-1H-1,2,3-triazole-4-carboxylate (**20**)

Yellowish oil, yield: 50%, ^{1}H NMR (400 MHz, CDCl$_3$) δ 8.26 (s, 1H, 28-CH), 5.21 (ds, 2H, 29-CH$_2$), 5.09 (ds, 2H, 12β-H, 12'β-H), 5.05–4.98 (m, 1H, 3β-H), 4.92 (ds, 2H. 7β-H, 7'β-H), 4.74–4.66 (m, 1H, 3'β-H), 3.67 (ds, 6H, 25-CH$_3$, 25'-CH$_3$), 2.15 (s, 3H, 7'α-OCOCH$_3$), 2.12 (s, 3H, 12α-OCOCH$_3$), 2.09 (s, 3H, 12'α-OCOCH$_3$), 0.94 (s, 3H, 19-CH$_3$), 0.93 (s, 3H, 19'-CH$_3$), 0.82 (d, *J*=6.4 Hz, 3H, 21'-CH$_3$), 0.80 (d, *J*=6.3 Hz, 3H, 21-CH$_3$) 0.73 (ds, 6H, 18-CH$_3$, 18'-CH$_3$). ^{13}C NMR (101 MHz, CDCl$_3$) δ: 174.6 (C-24), 174.5 (C-24'), 170.7 (12α-O$\underline{C}$OCH$_3$), 170.4 (7'α-O$\underline{C}$OCH$_3$), 170.2 (12'α-O$\underline{C}$OCH$_3$), 165.1 (C-30), 160.0 (CO-26), 141.1 (C-27), 129.0 (C-28), 75.8 (C-12), 75.6 (C-12', C-3), 75.3 (C-3'), 70.5 (C-7'), 51.5 (C-25, C-25'), 51.2 (C-29), 49.3, 47.5, 47.3, 45.0, 43.3, 42.0, 40.8, 37.7, 35.7, 34.7, 34.6, 34.6, 34.5, 34.4, 34.2, 34.2, 32.3, 31.2, 30.9, 30.9, 30.8, 30.7, 28.8, 27.3, 27.1, 27.0, 26.7, 26.6, 25.8, 25.8, 25.5, 23.4, 23.1 (C-19), 22.8, 22.4 (C-19'), 21.6 (12'α-OCO$\underline{C}$H$_3$), 21.5 (12α-OCO$\underline{C}$H$_3$), 21.4

(7'α-OCO$\underline{C}H_3$), 17.5 (C-21, C-21'), 14.2, 12.4 (C-18), 12.2 (C-18'). FT-IR (KBr, cm^{-1}) ν_{max}: 2951, 2872, 1736, 1450, 1378, 1245, 1042, 1022, 969. ESI-MS (MeOH) *m/z* (%): 1113 [M+Na]$^+$, 1129 [M+K]$^+$.

1.9. 24-methoxy-24-oxo-7α,12α-diacetoxy-5β-cholan-3β-yl[1-(24-metoxy-24-oxo-7α,12α-diacetoxy-5β-cholan-3β-yl)]-1H-1,2,3-triazole-4-carboxylate (**21**)

Yellowish oil, yield: 70%, ^{1}H NMR (400 MHz, $CDCl_3$) δ 8.28 (s, 1H, 28-CH), 5.21 (ds, 2H, 29-CH_2), 5.09 (ds, 2H, 12β-H, 12'β-H), 4.94–4.86 (m, 3H, 3β-H, 7β-H, 7'β-H), 4.74–4.66 (m, 1H, 3'β-H), 3.67 (s, 6H, 25-CH_3, 25'-CH_3), 2.21 (s, 3H, 7α-OCOCH_3), 2.14 (s, 3H, 7'α-OCOCH_3), 2.09 (s, 3H, 12'α-OCOCH_3), 2.08 (s, 3H, 12α-OCOCH_3), 0.95 (s, 3H, 19-CH_3), 0.93 (s, 3H, 19'-CH_3), 0.82 (d, *J*=6.3 Hz, 3H, 21'-CH_3), 0.81 (d, *J*=6.3 Hz, 3H, 21-CH_3), 0.73 (s, 6H, 18-CH_3, 18'-CH_3). ^{13}C NMR (101 MHz, $CDCl_3$) δ: 174.5 (C-24), 174.5 (C-24'), 170.8 (7α-O$\underline{C}$OCH$_3$), 170.6 (12α-O$\underline{C}$OCH$_3$), 170.4 (7'α-O$\underline{C}$OCH$_3$), 170.2 (12'α-O$\underline{C}$OCH$_3$), 165.1 (C-30), 160.1 (CO-26), 141.1 (C-27), 129.2 (C-28), 75.5 (C-12, C-12'), 75.3 (C-3, C-3'), 70.5 (C-7, C-7'), 51.5 (C-25, C-25'), 51.2 (C-29), 47.3, 47.3, 45.1, 45.0, 43.4, 43.3, 41.1, 40.8, 37.7, 37.7, 34.7, 34.6, 34.5, 34.5, 34.4, 34.4, 34.2, 31.3, 31.2, 30.9, 30.8, 30.7, 29.1, 28.8, 27.1, 26.8, 26.7, 25.7, 25.5, 22.8, 22.6 (C-19), 22.4 (C-19'), 21.8 (7α-OCO$\underline{C}H_3$), 21.7 (12α-OCO$\underline{C}H_3$), 21.6 (12'α-OCO$\underline{C}H_3$), 21.4 (7'α-OCO$\underline{C}H_3$), 17.5 (C-21, C-21'), 12.2 (C-18, C-18'). FT-IR (KBr, cm^{-1}) ν_{max}: 2952, 2873, 1736, 1378, 1247, 1042, 1022, 963. ESI-MS (MeOH) *m/z* (%): 1055 [M+Na]$^+$, 1071 [M+K]$^+$, 1067 [M+Cl]$^-$.

2. PM5 Calculations

The PM5 semiempirical calculations were performed using the WinMopac 2003 program.

3. Biological Activity

3.1. Human Red Blood Cells

Human red blood cell (RBC) concentrates (hematocrit 65%) were purchased from the blood bank in Poznan according to the bilateral agreement no ZP/2867/D/21 signed between the Reginal Blood Center in Poznań and Adam Mickiewicz University in Poznań. The RBC suspension was washed three times (960 × g, 10 min, +4 °C) in 7.4 pH phosphate-buffered saline (PBS PBS: 137 mM NaCl, 2.7 mM KCl, 10 mM Na_2HPO_4, 1.76 mM KH_2PO_4) supplemented with 10 mM glucose. After washing, the cells were suspended in PBS buffer at 1.65 × 109 cells/mL (Ht = 15%), stored at + 4 °C and used within 5 h.

3.2. Hemolysis Assay

The compounds' cytotoxicity was determined by a standard hemolytic assay, as previously reported. Briefly, human RBC (1.65 × 108 cells/mL, hematocrit 1.5%) were incubated in PBS (7.4 pH) supplemented with 10 mM glucose and containing compounds tested at a 0.1 mg/mL concentration for

60 min at 37 °C under gentle shaking. Samples with RBC incubated in PBS without compounds tested were taken as the negative control. Samples with RBC incubated in ice-cold deionized water without compounds tested were taken as the positive control. Each sample was prepared in triplicate and the experiments were repeated three times with RBC from different donors. After incubation, RBC suspensions were centrifuged (3000 rpm, 10 min, +4 °C), and the degree of compounds-induced hemolysis was assessed by measuring the absorbance value of the supernatant at λ = 540 nm in a BioMate™ 160 UV–Vis spectrophotometer (Thermo Scientific, Waltham, MA, USA).The results were expressed as the percentage of hemolysis, which was determined using the formula:

hemolysis (%) = (sample absorbance/ positive control absorbance) × 100 (*Eqn. 1*).

The results are presented as a mean value (±SD) of three independent experiments (n = 12). A hemolysis degree of less than 5% indicated very weak hemolytic activity of the compounds tested. A hemolysis degree higher than 10% showed significant hemolytic activity of compounds at the given concentration.

4. Molecular Docking

Both crystal structures (PDB: 3U2D and 3VMT) were downloaded from the RCSB protein bank. Docking was performed using Maestro version 13.4.134, Release 2022-4, Schrodinger software. The optimized three-dimensional structures for the compounds were determined by LigPrep (OPLS3) and the protonation state at pH 7.4 ± 2.0 using Epik. Protein Preparation Wizard was used to evaluate appropriate amino acid ionization states, to check the steric clashes and to assign bond ordering. The compounds were docked by Induced Fit Docking (IFD) with XP (extra precision) with the OPLS3 force field. A grid box size of 15 Å was centered on a co-crystallized compound. Images were prepared using Maestro.

5. NMR data and ESI-MS spectra of bioconjugates (**13-21**)

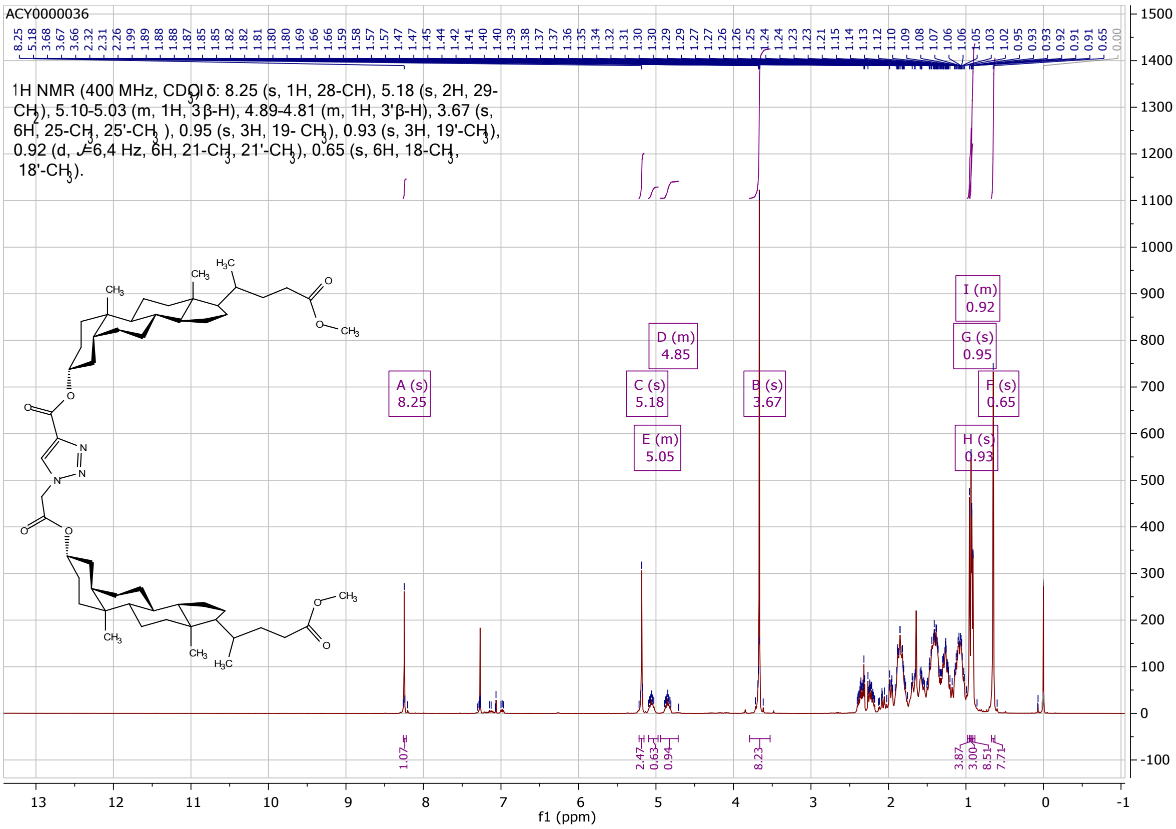

Figure S1.^{1}H NMR spectrum of conjugate (13).

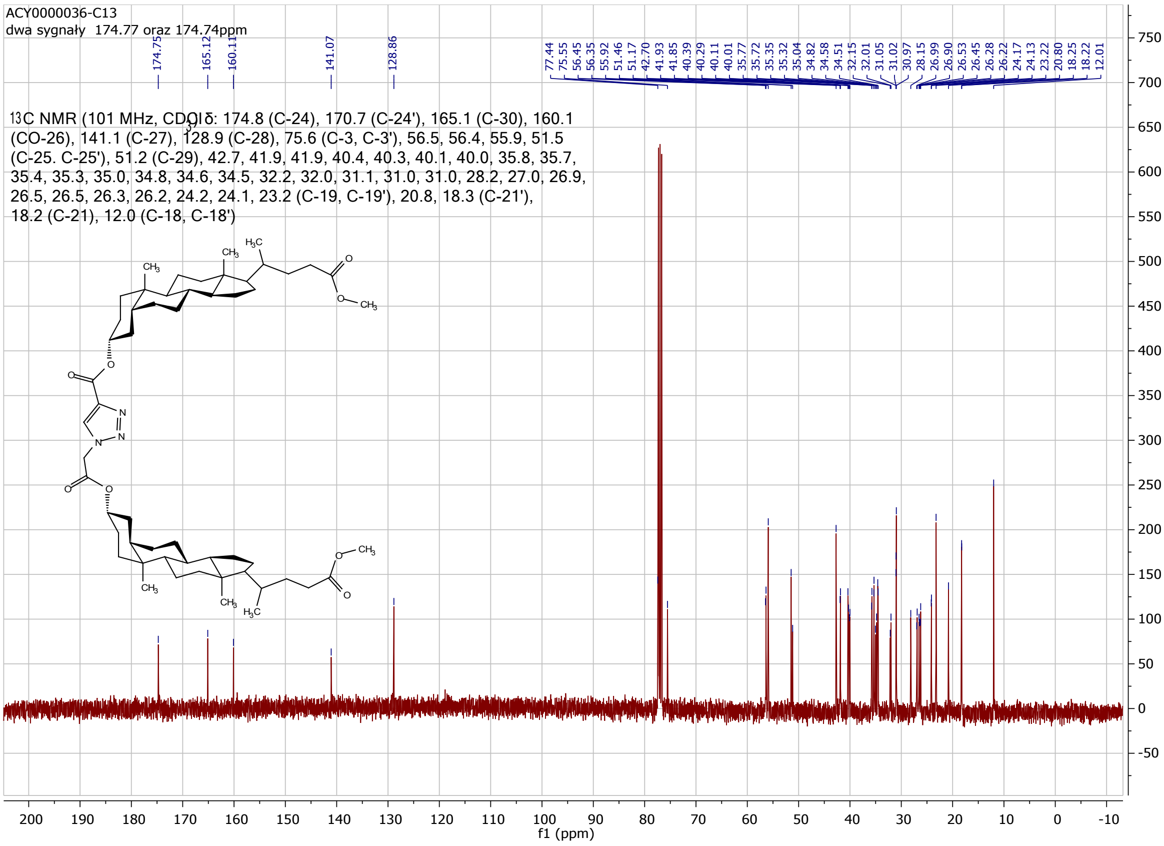

Figure S2. ^{13}C NMR spectrum of conjugate (13).

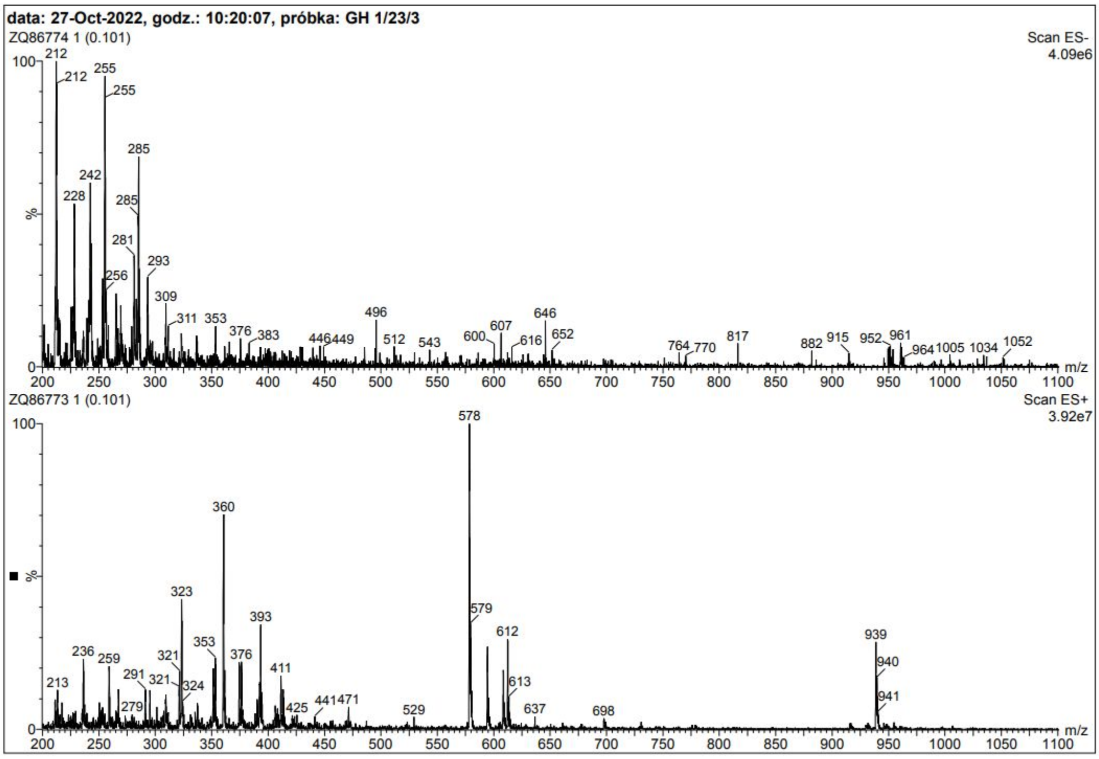

Figure S1. ESI-MS spectrum of conjugate (13).

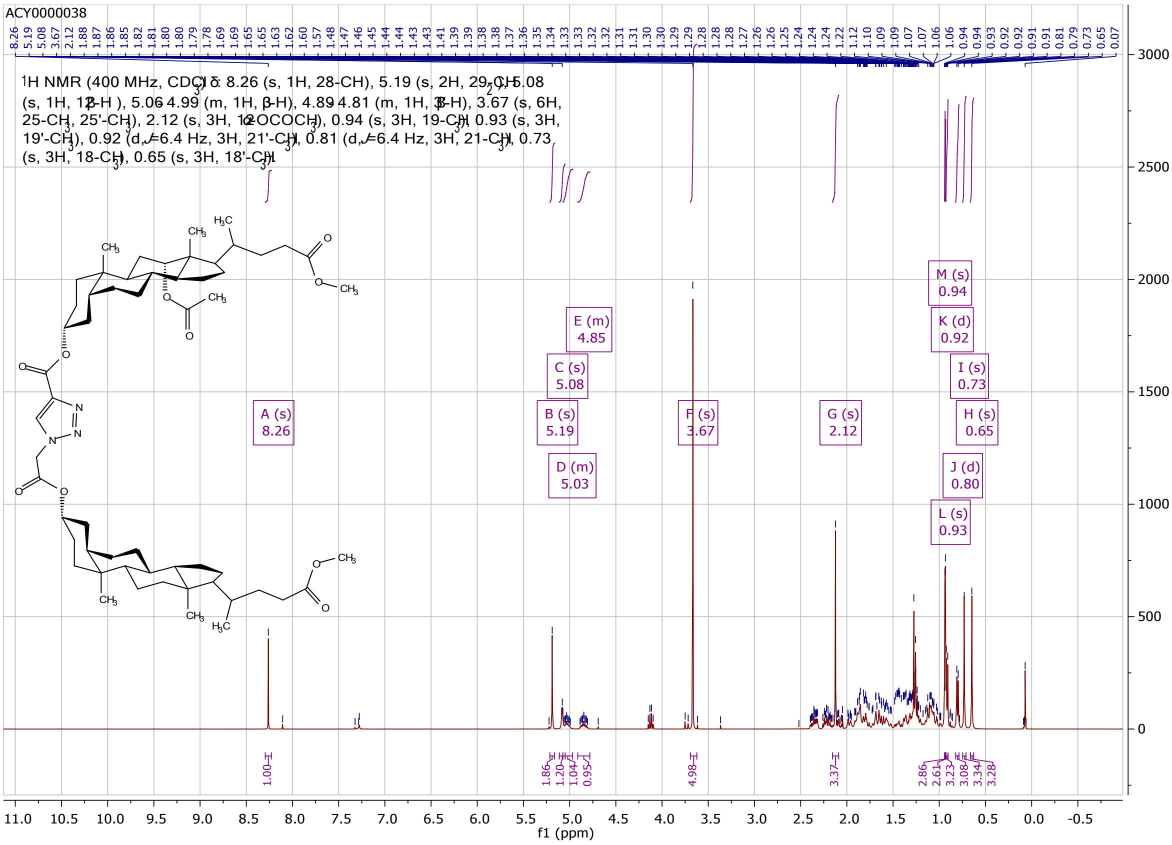

Figure S2. ^{1}H NMR spectrum of conjugate (14).

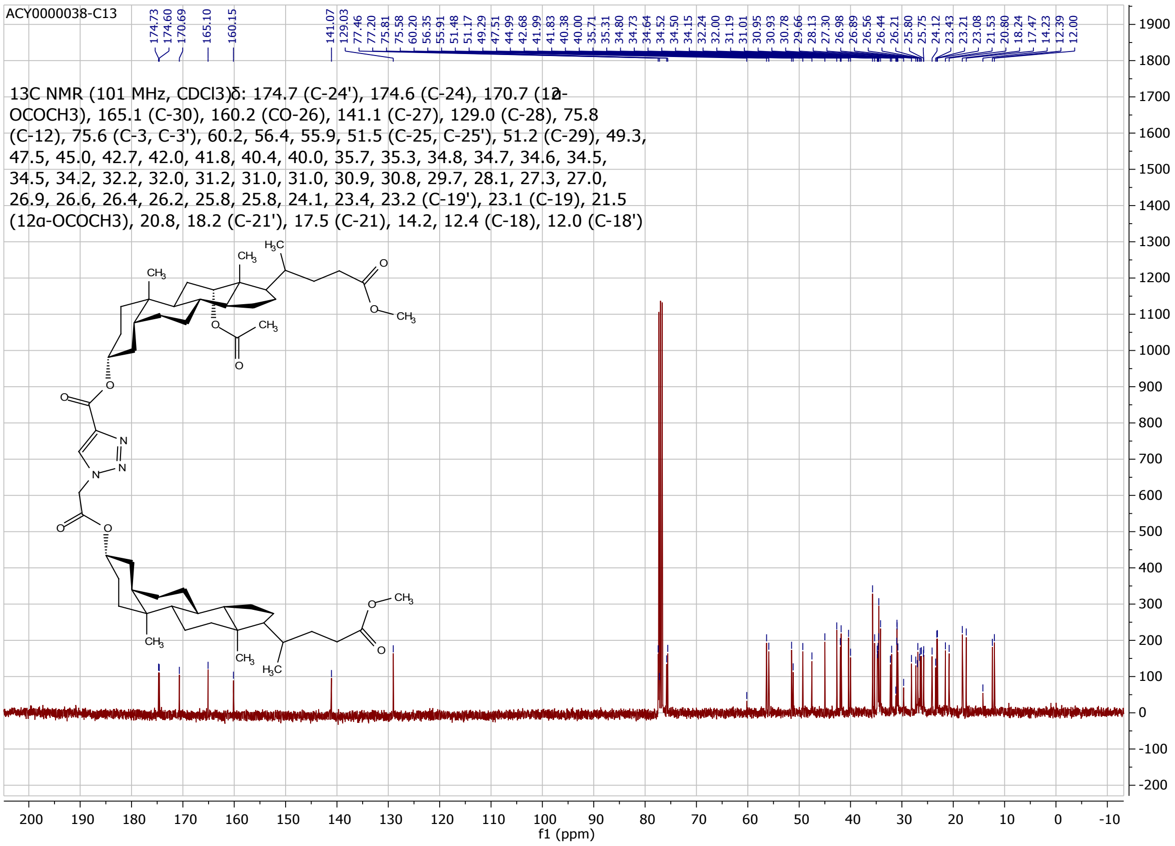

Figure S3. ^{13}C NMR spectrum of conjugate (14).

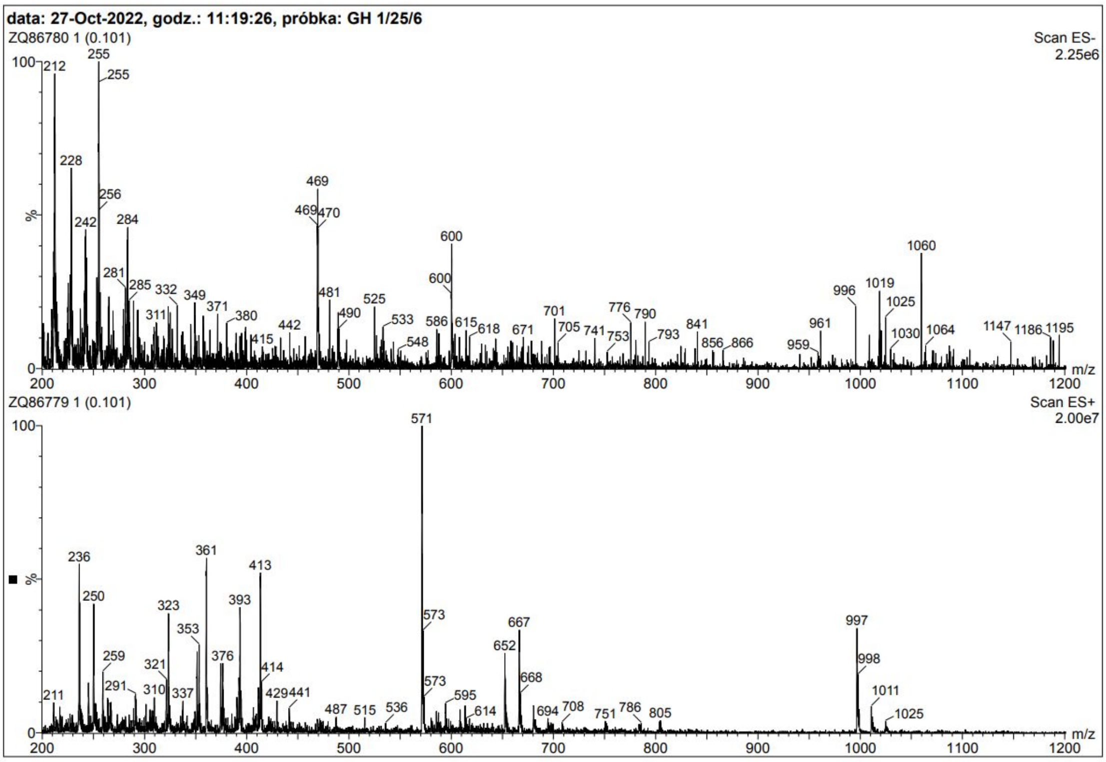

Figure S4. ESI-MS spectrum of conjugate (14).

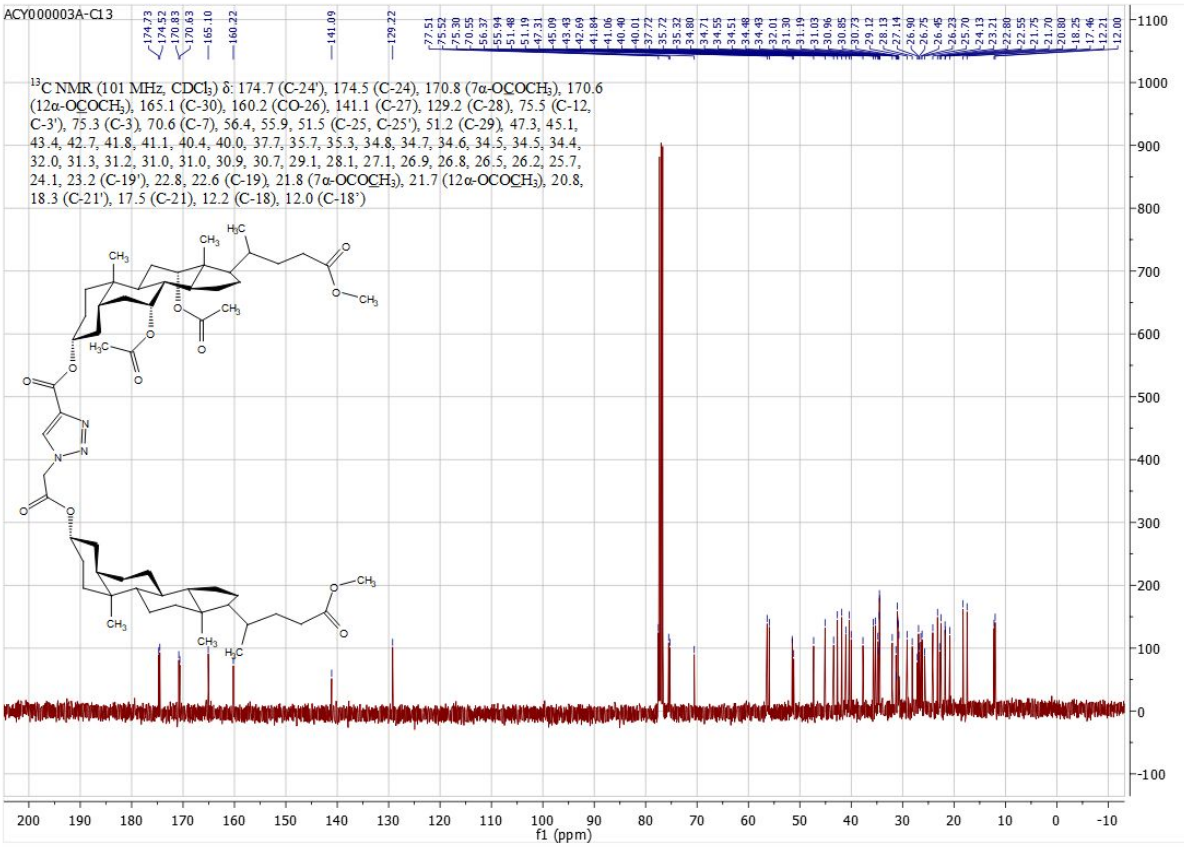

Figure S5. ^{1}H NMR spectrum of conjugate (15).

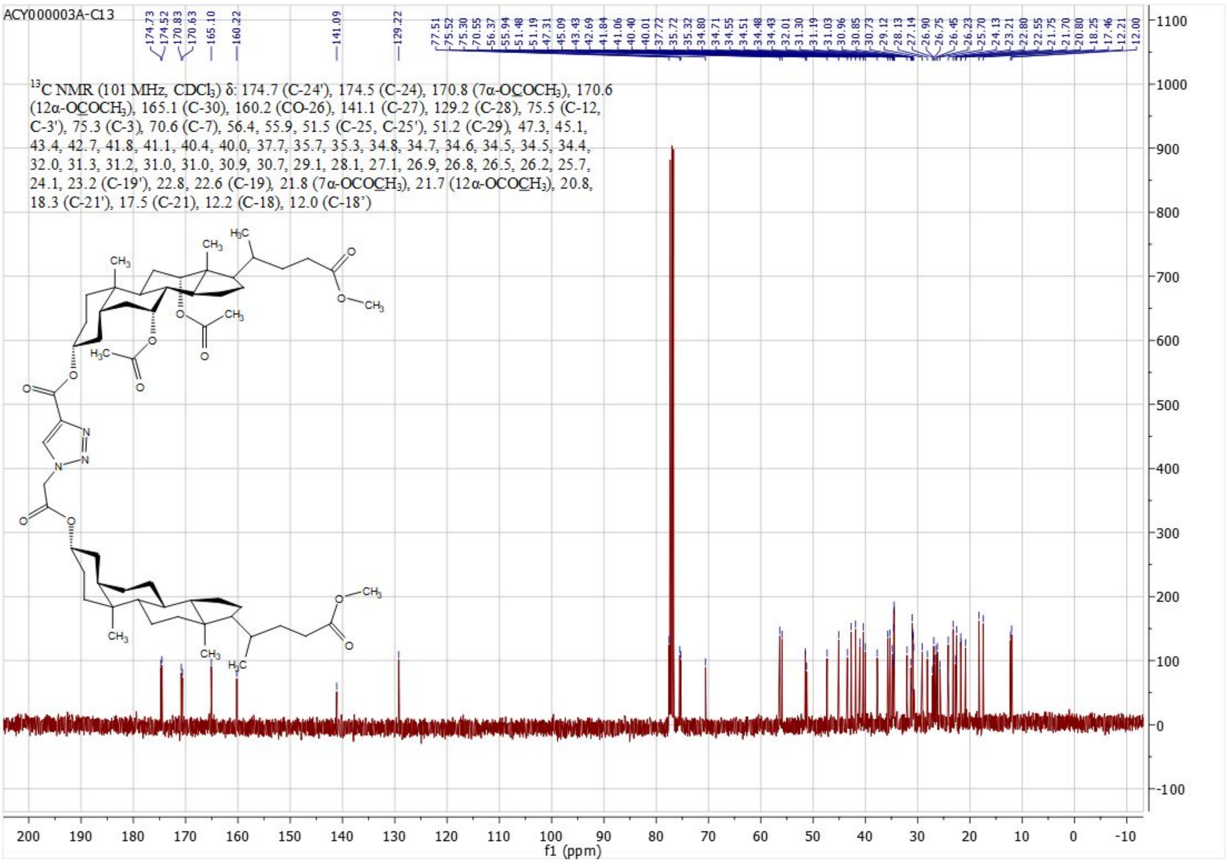

Figure S6. ^{13}C NMR spectrum of conjugate (15).

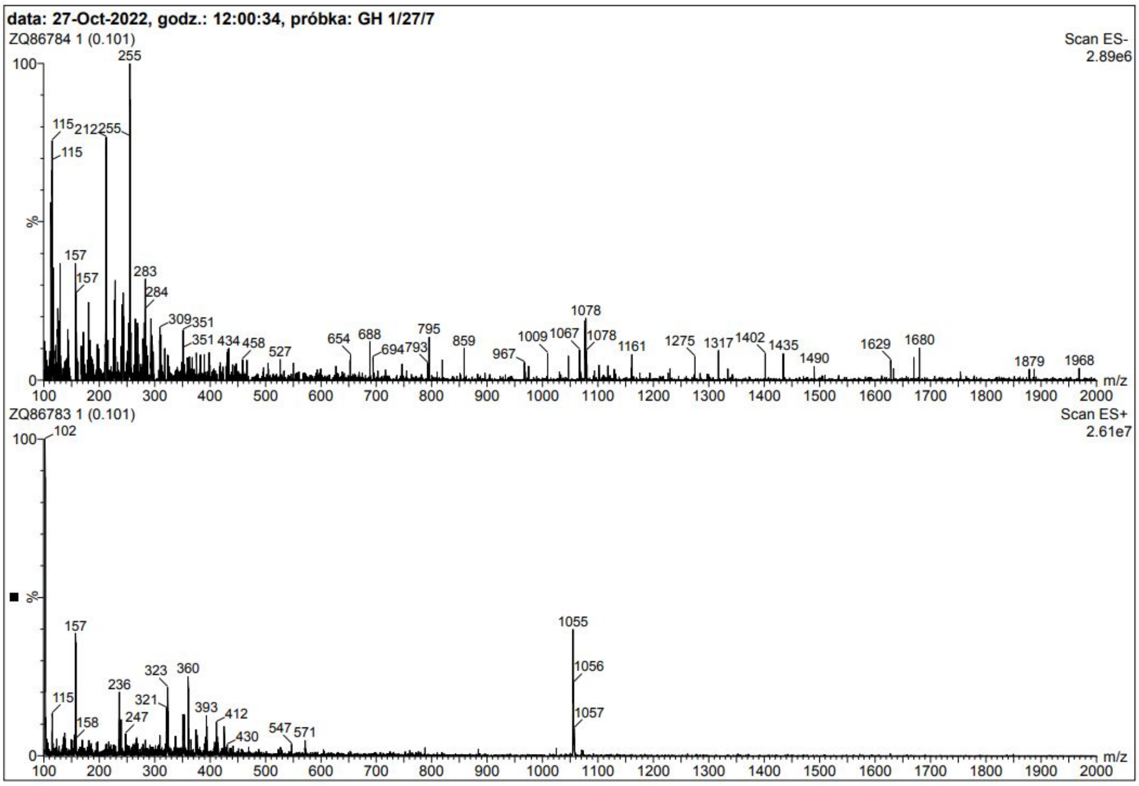

Figure S7. ESI-MS spectrum of conjugate (15).

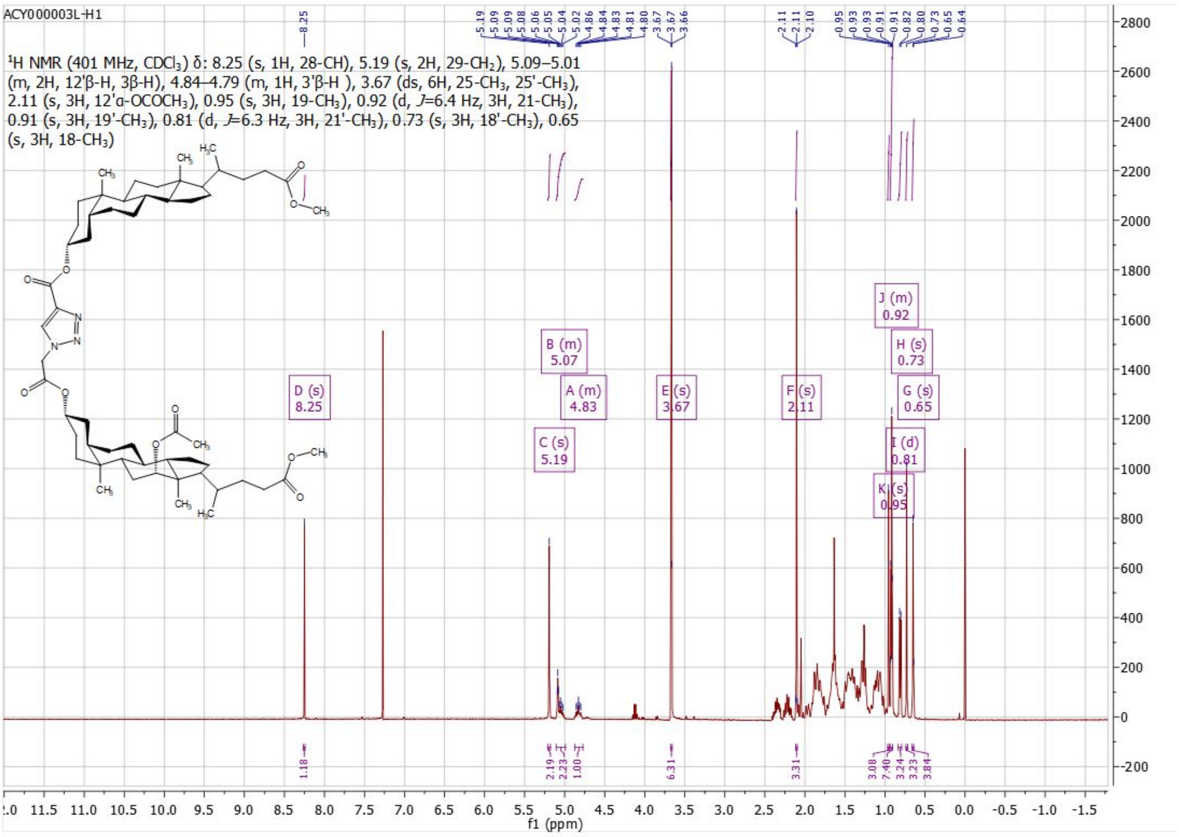

Figure S8. ^{1}**H NMR spectrum of conjugate (16).**

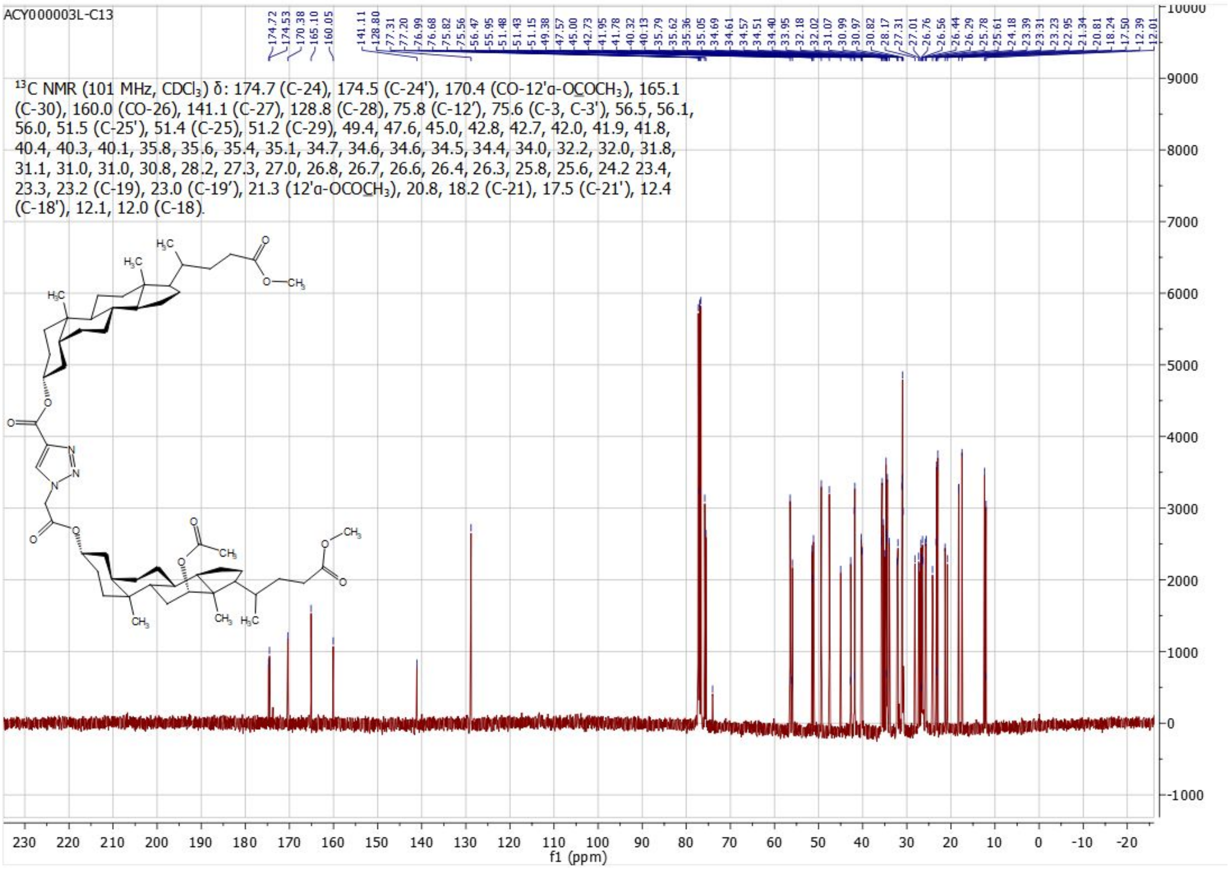

Figure S9. ^{13}C NMR spectrum of conjugate (16).

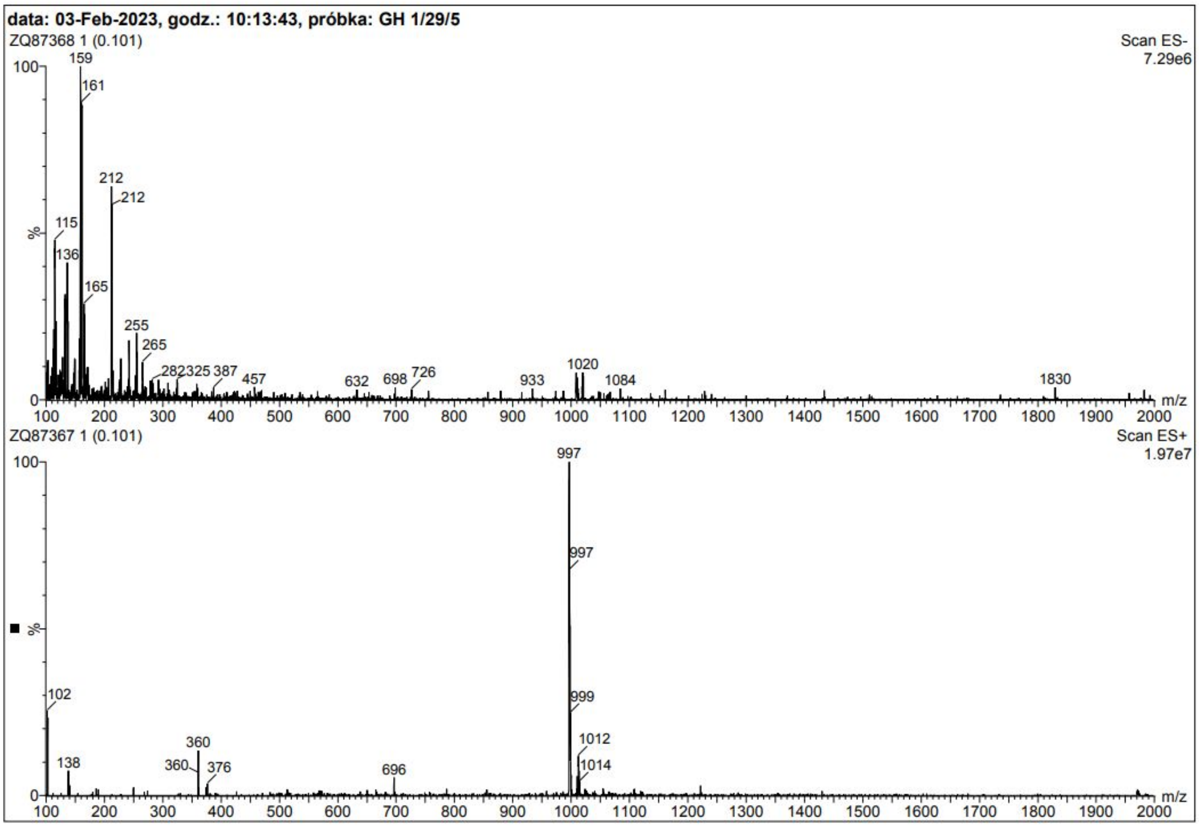

Figure S10. ESI-MS spectrum of conjugate (16).

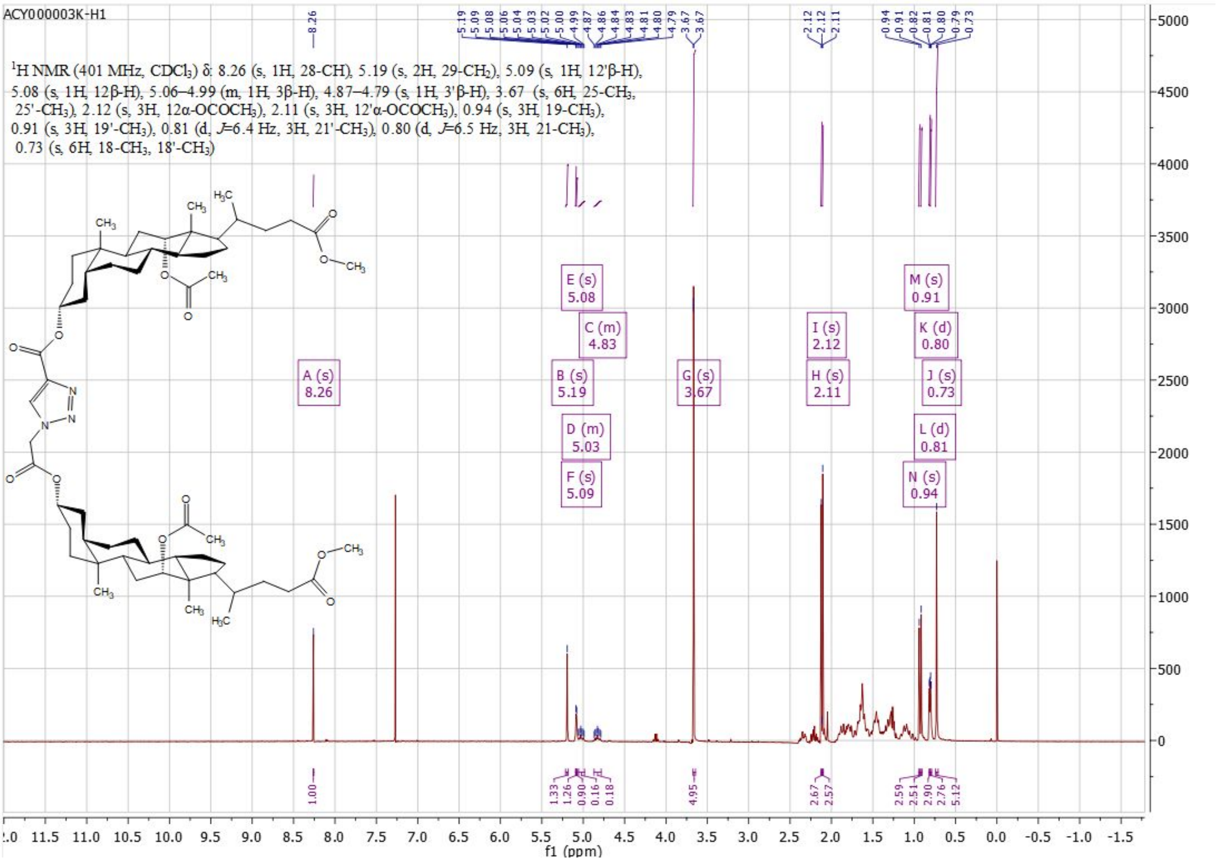

Figure S13. ^{1}H NMR spectrum of conjugate (17).

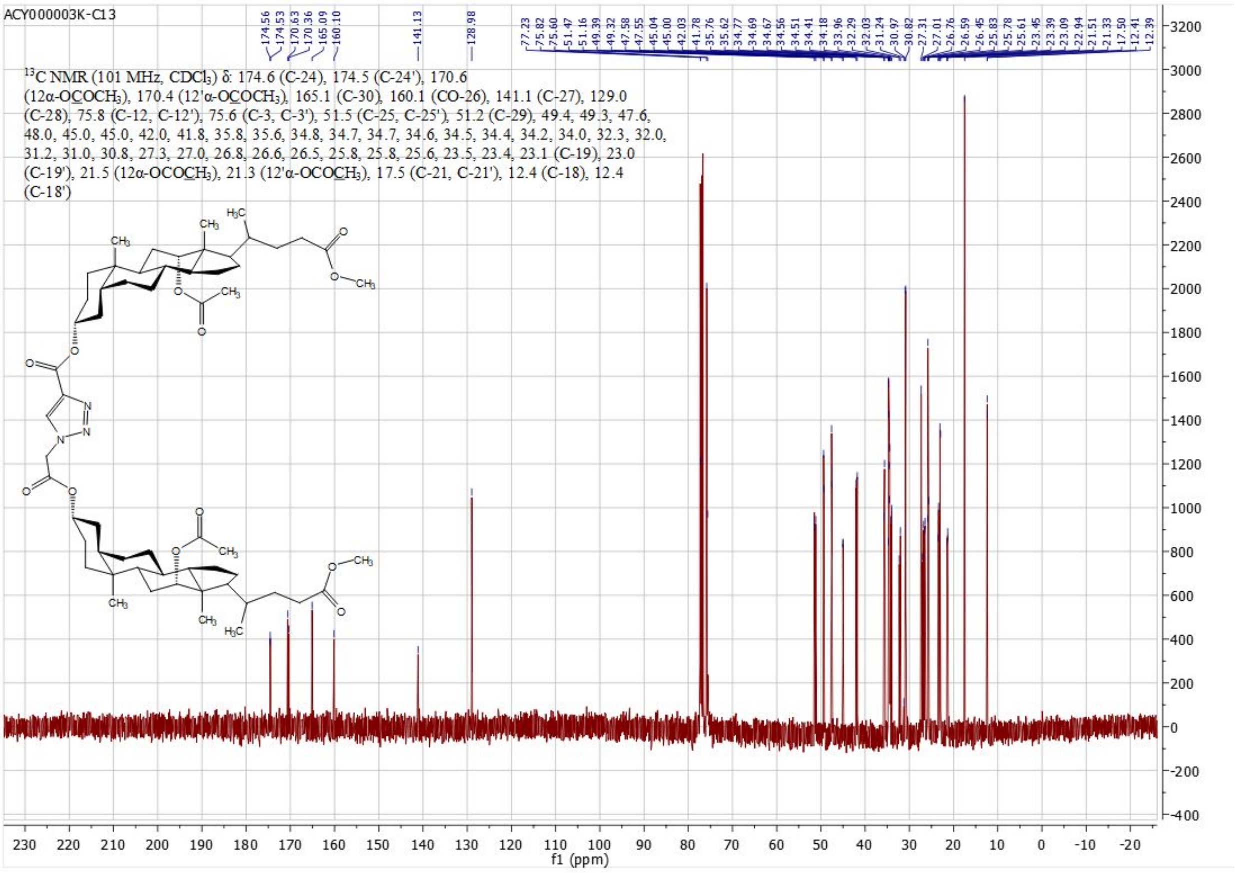

Figure S14. ^{13}C NMR spectrum of conjugate (17).

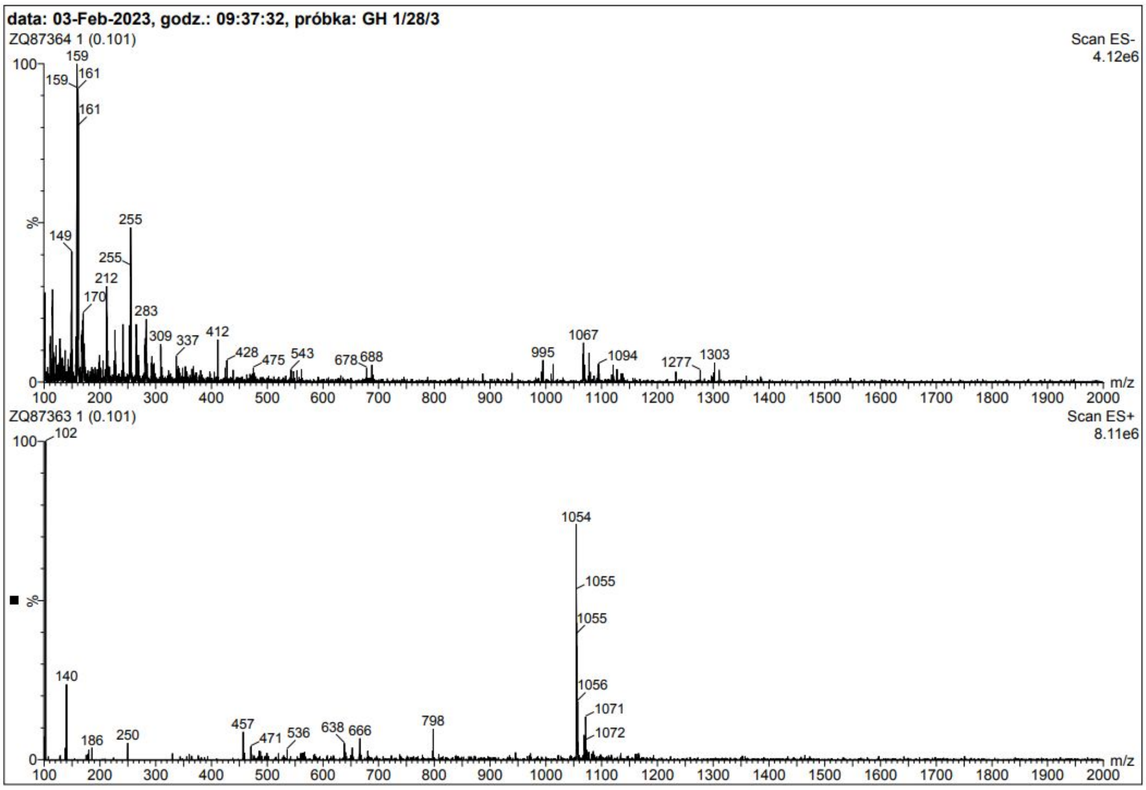

Figure S15. ESI-MS spectrum of conjugate (17).

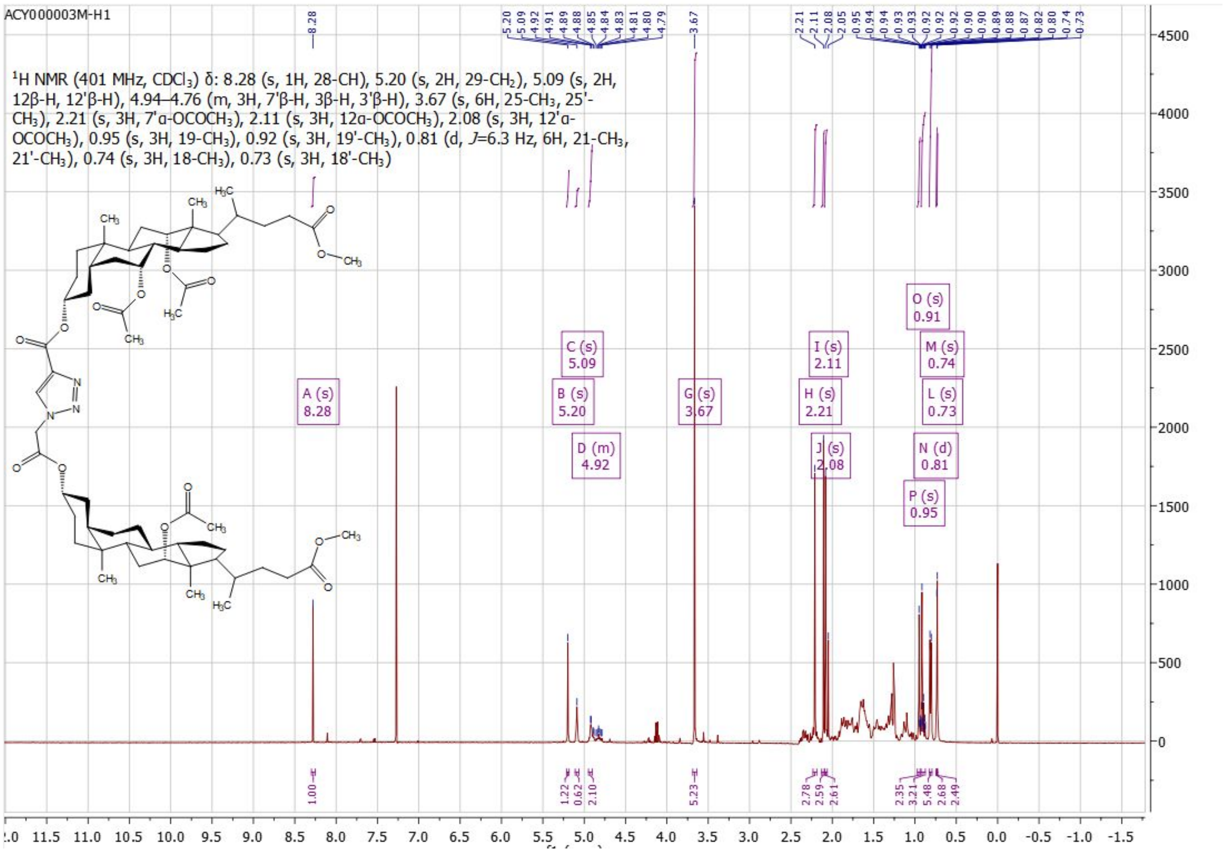

Figure S16. ¹H NMR spectrum of conjugate (18).

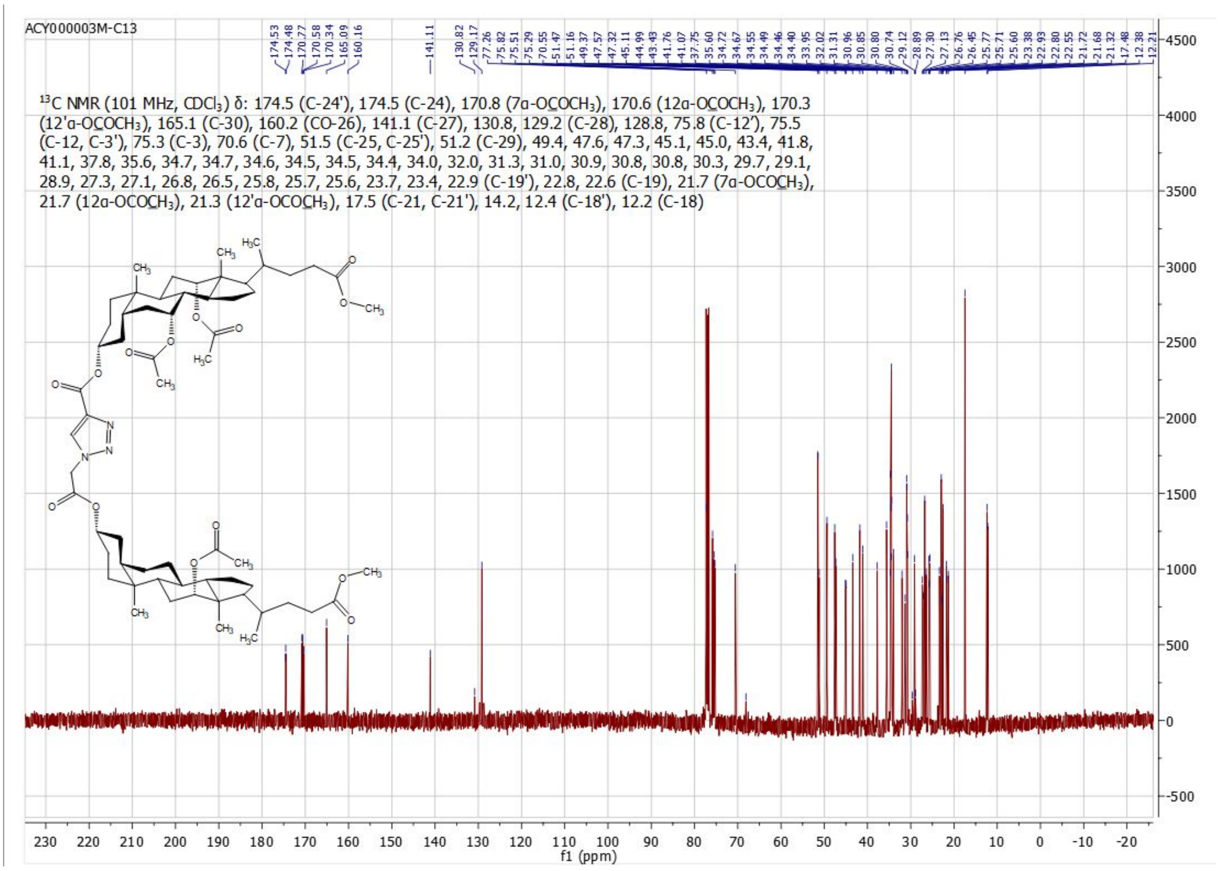

Figure S17. ^{13}C NMR spectrum of conjugate (18).

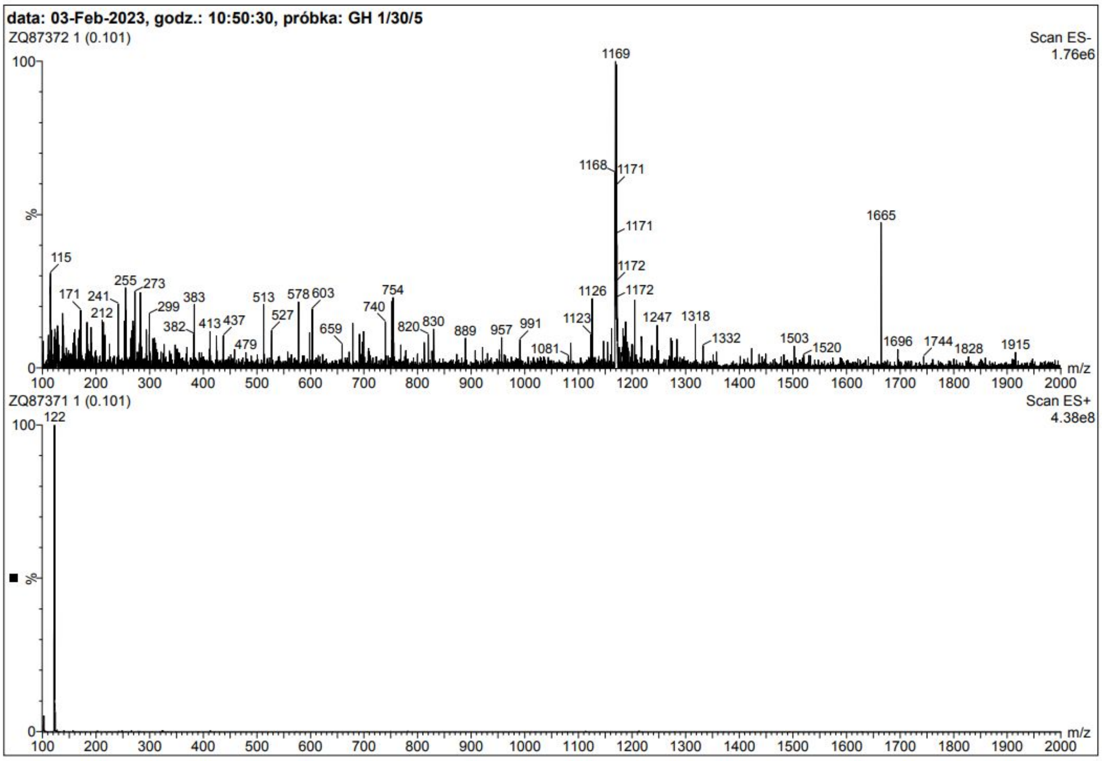

Figure S18. ESI-MS spectrum of conjugate (18).

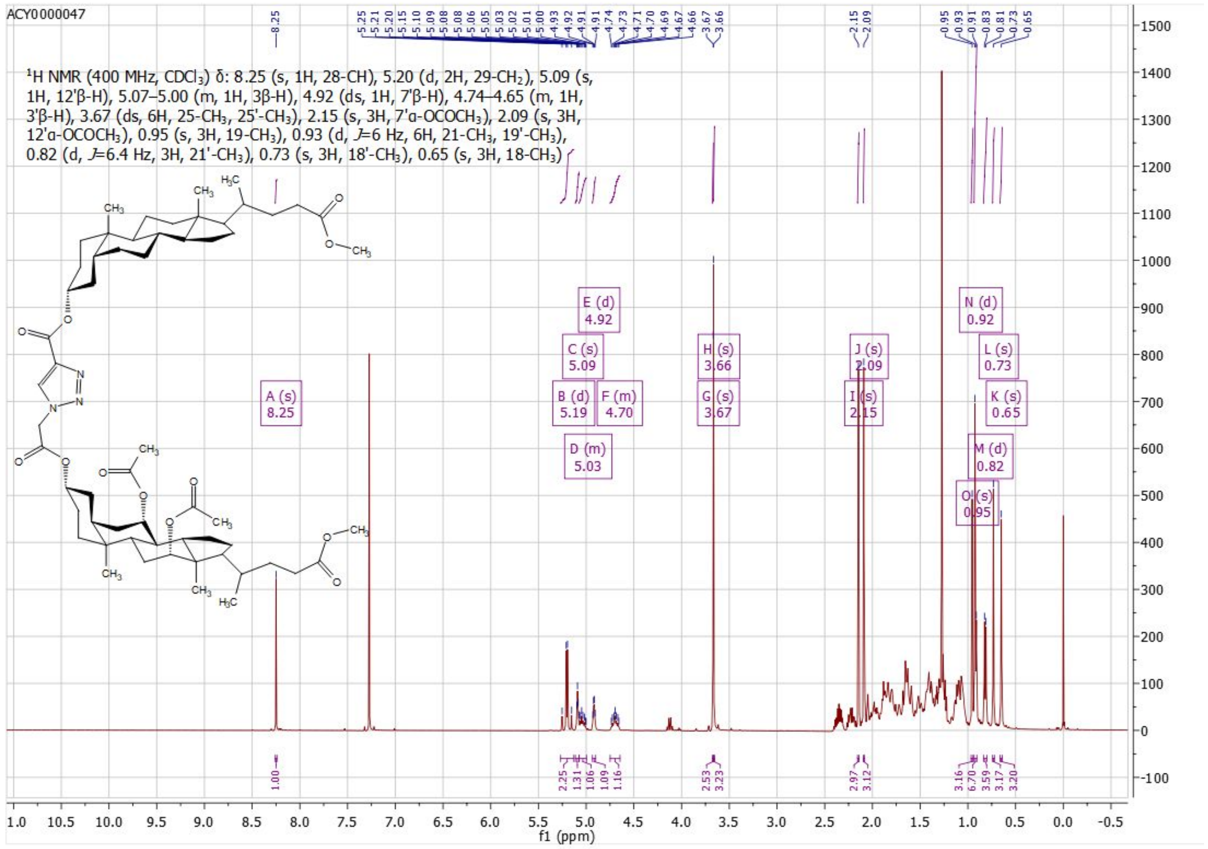

Figure S19. ¹H NMR spectrum of conjugate (19).

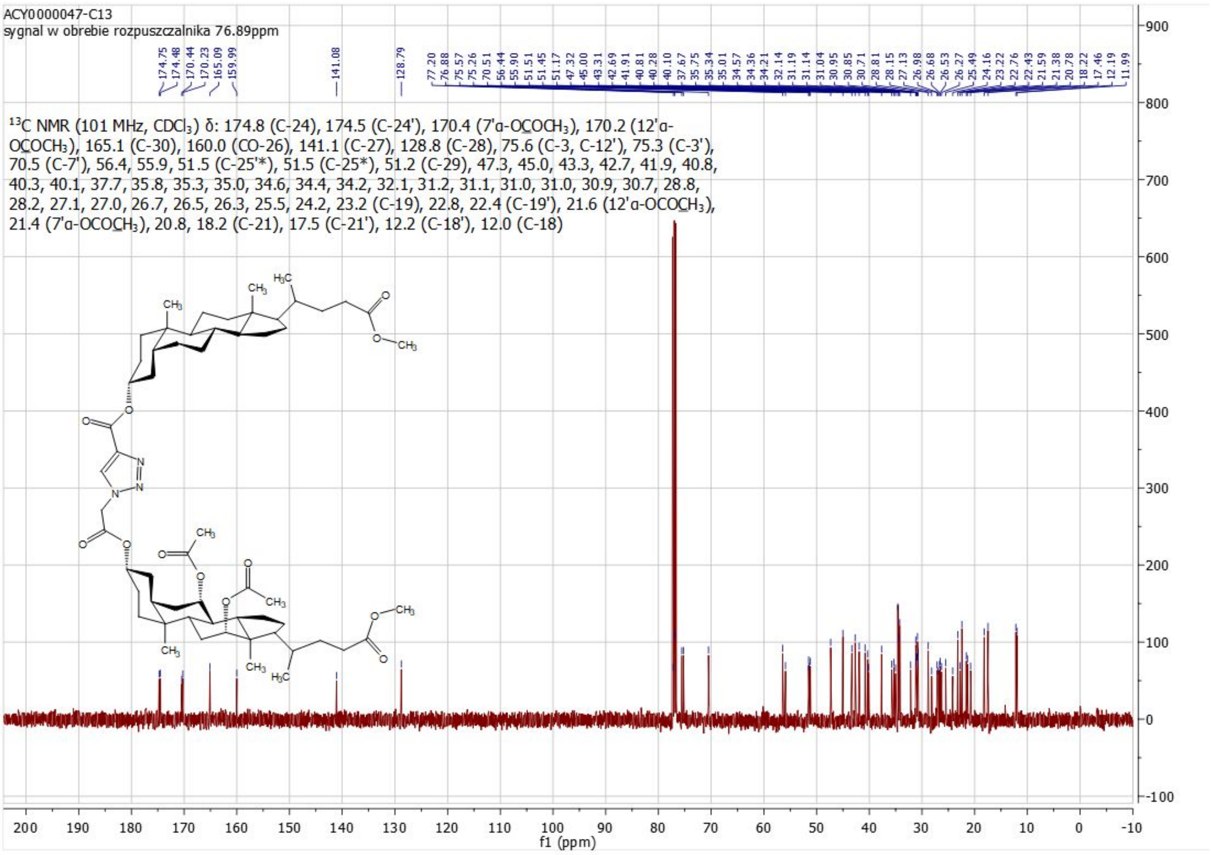

Figure S20. ¹³C NMR spectrum of conjugate (19).

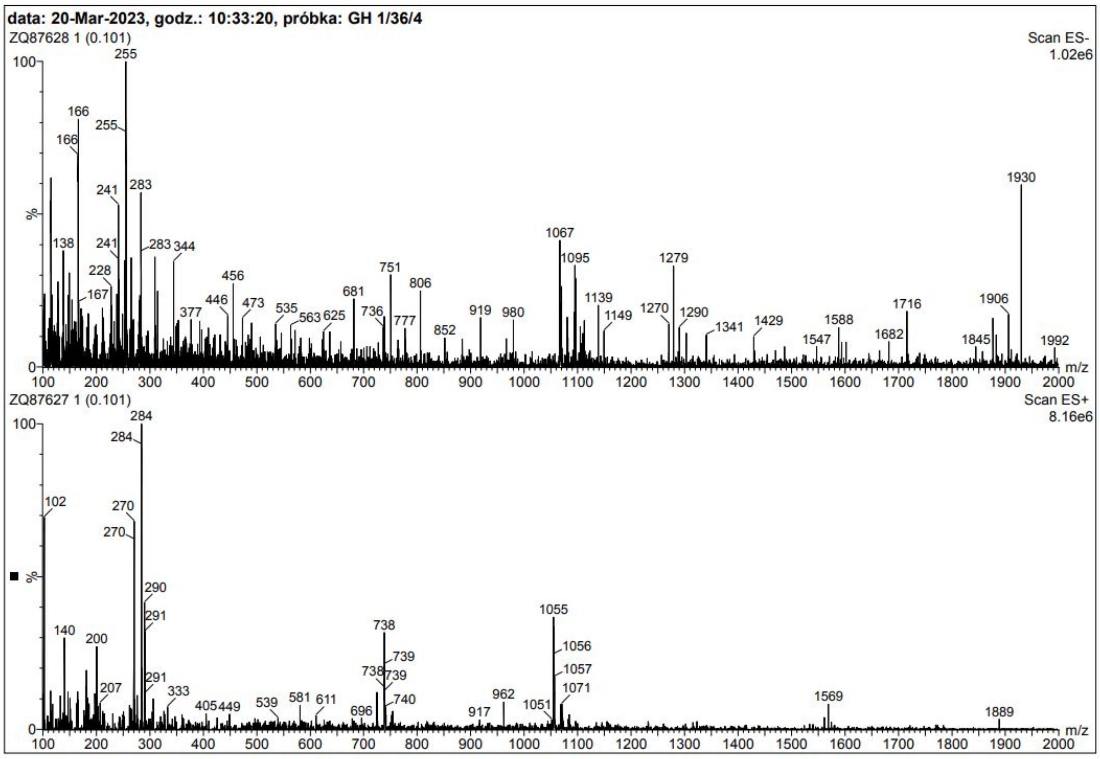

Figure S21. ESI-MS spectrum of conjugate (19).

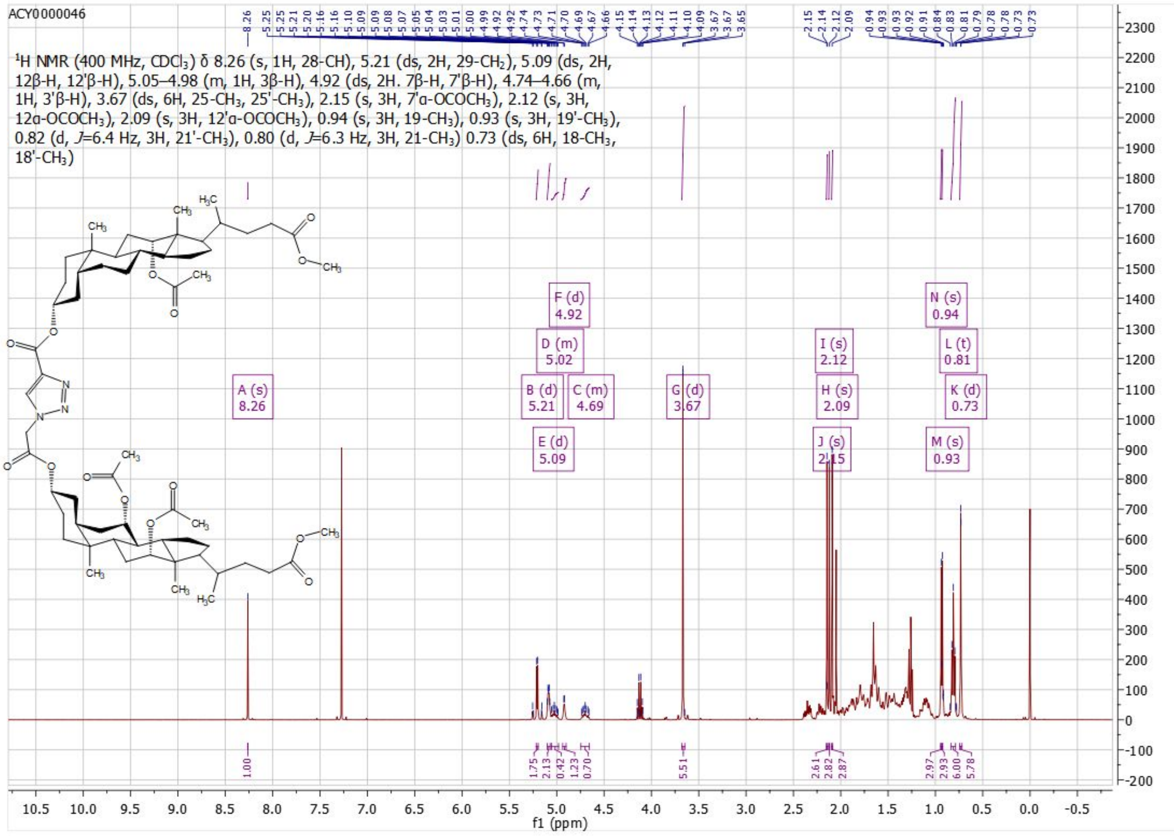

Figure S22. ¹H NMR spectrum of conjugate (20).

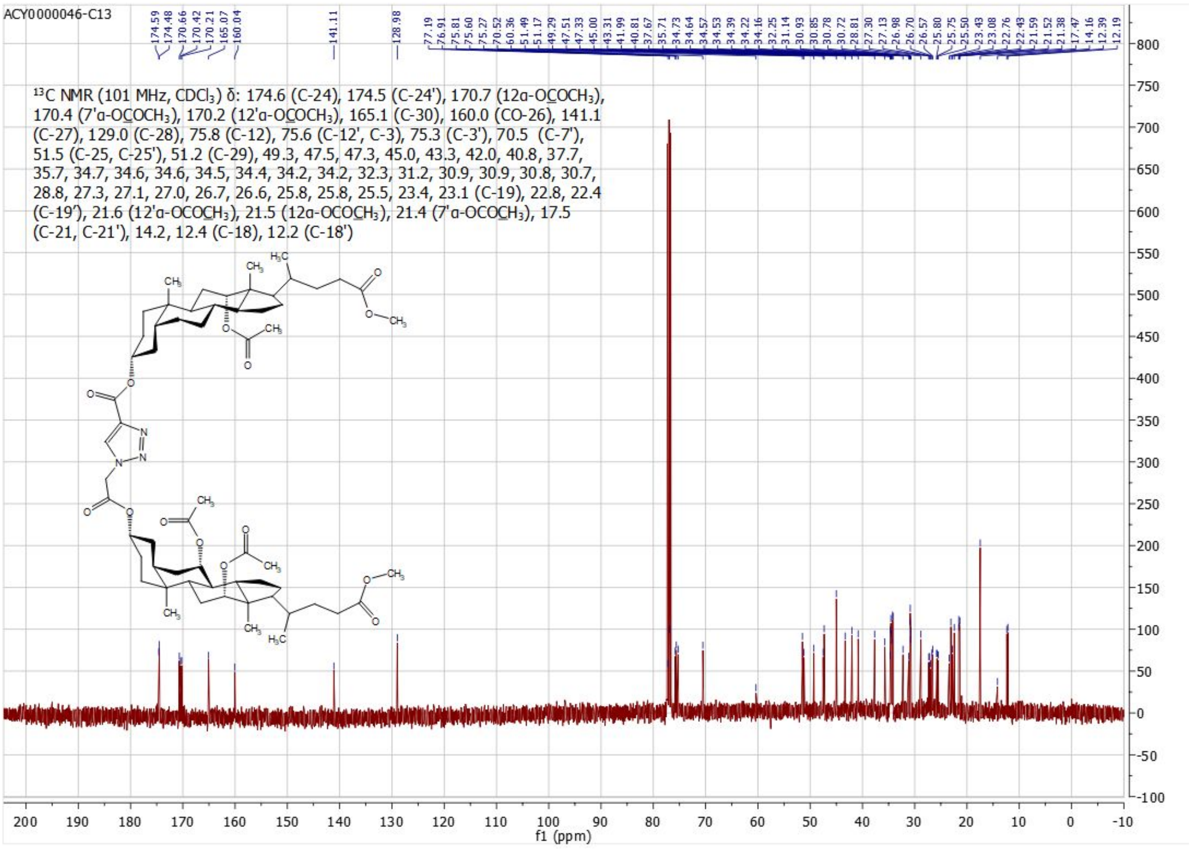

Figure S23. ^{13}C NMR spectrum of conjugate (20).

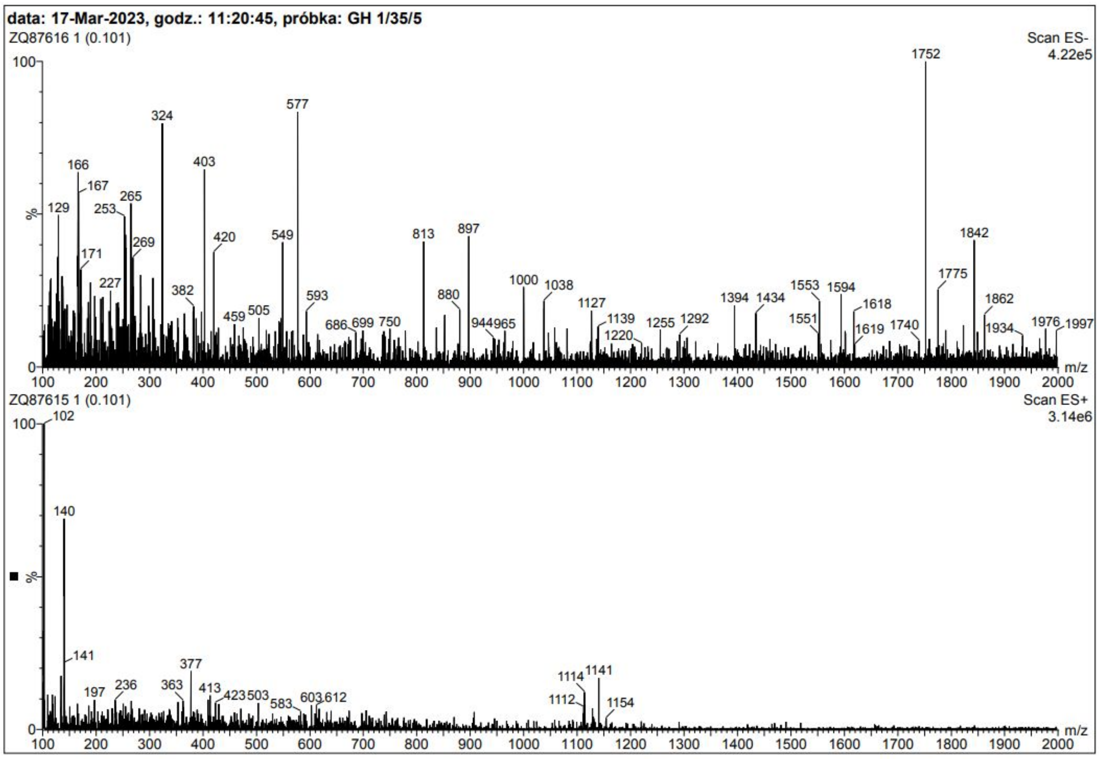

Figure S24. ESI-MS spectrum of conjugate (20).

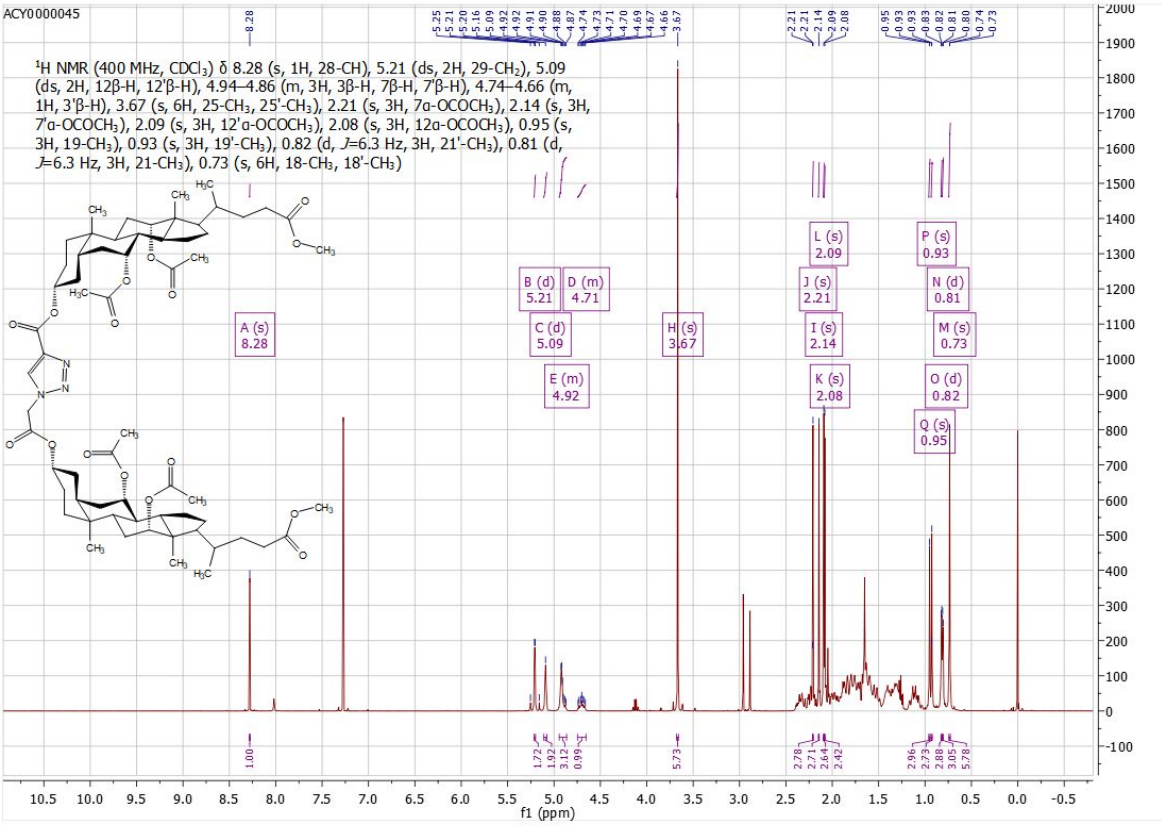

Figure S25. ^{1}H NMR spectrum of conjugate (21).

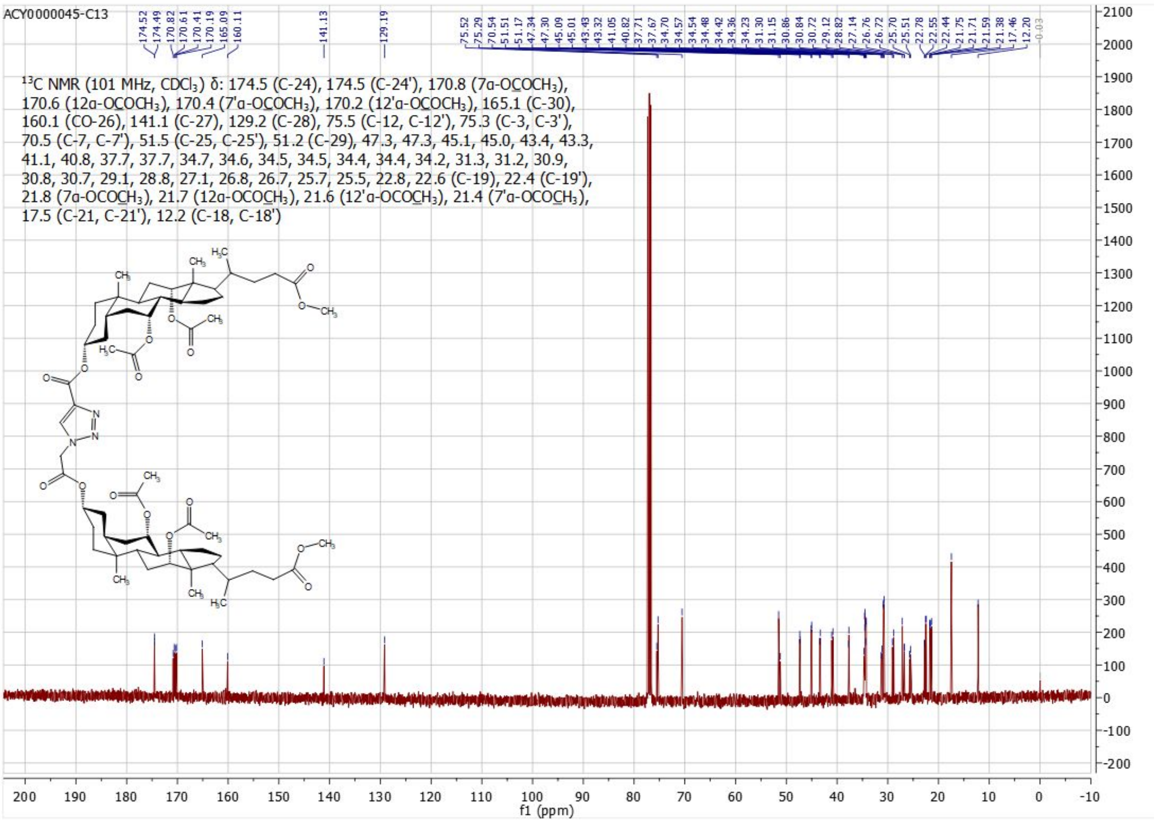

Figure S26. ^{13}C NMR spectrum of conjugate (21).

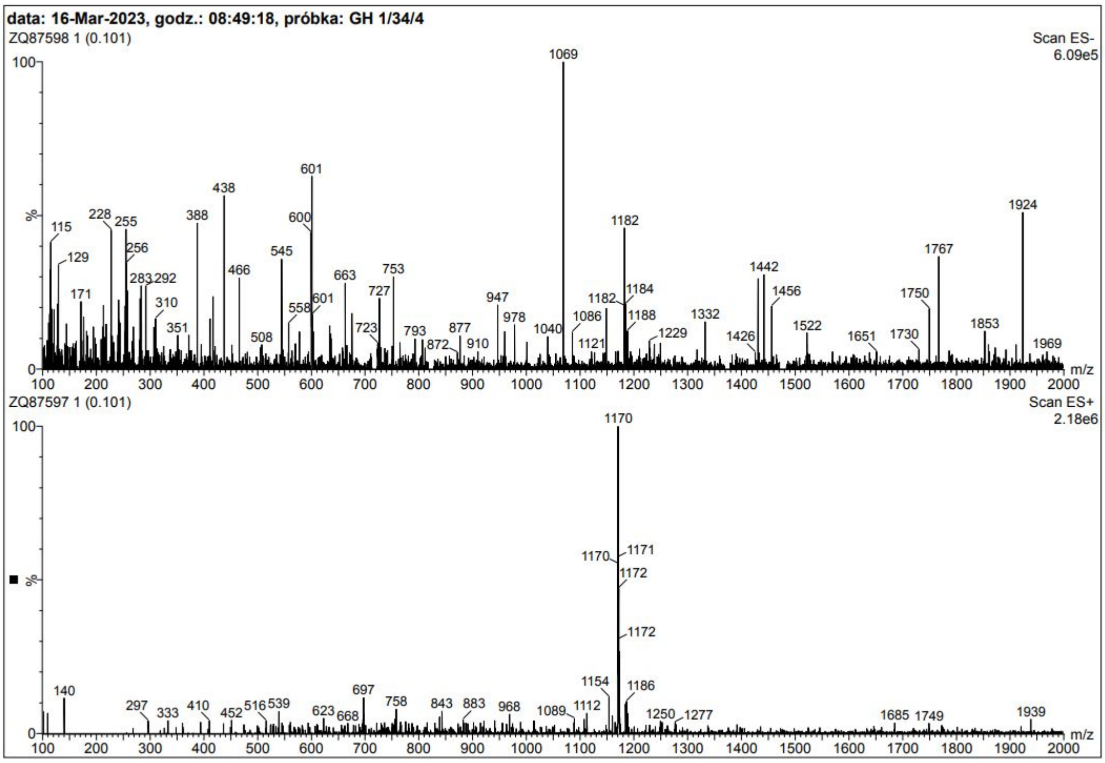

Figure S27. ESI-MS spectrum of conjugate (21).

References

[1]. Selick, H. E.; Beresford, A. P.; Tarbit, M. H. The emerging importance of predictive ADME simulation in drug discovery. *Drug Discov. Today* **2002**, 7(2), 109-116. DOI: 10.1016/S1359-6446(01)02100-6.

[2]. Sheng, R.; Luo, T.; Li, H.; Sun, J.; Wang, Z.; Cao, A. 'Click' synthesized sterol-based cationic lipids as gene carriers, and the effect of skeletons and headgroups on gene delivery. *Bioorg. Med. Chem.* **2013**, 21(21), 6366-6377. DOI: 10.1016/j.bmc.2013.08.047

[3]. D'Amore, C.; Di Leva, F. S.; Sepe, V.; Renga, B.; Del Gaudio, C.; D'Auria, M. V.; Zampella, A.; Fiorucci, S.; Limongelli, V. Design, synthesis, and biological evaluation of potent dual agonists of nuclear and membrane bile acid receptors. *J. Med. Chem.* **2014**, 57(3), 937–954. DOI: 10.1021/jm401873d
